# Engineering a functional thyroid as a potential therapeutic substitute for hypothyroidism treatment: A systematic review

**DOI:** 10.3389/fendo.2022.1065410

**Published:** 2022-12-02

**Authors:** Lei Li, Qixuan Sheng, Huajin Zeng, Wei Li, Qiang Wang, Guanjun Ma, Ming Qiu, Wei Zhang, Chengxiang Shan

**Affiliations:** Department of Thyroid and Breast Surgery of Changzheng Hospital Affiliated with Naval Military Medical University, Shanghai, China

**Keywords:** thyroid, hypothyroidism, regeneration, organoid, tissue engineering

## Abstract

**Background:**

Hypothyroidism is a common hormone deficiency disorder. Although hormone supplemental therapy can be easily performed by daily levothyroxine administration, a proportion of patients suffer from persisting complaints due to unbalanced hormone levels, leaving room for new therapeutic strategies, such as tissue engineering and regenerative medicine.

**Methods:**

Electronic searches of databases for studies of thyroid regeneration or thyroid organoids were performed. A systematic review including both *in vitro* and *in vivo* models of thyroid regenerative medicine was conducted.

**Results:**

Sixty-six independent studies published between 1959 and May 1st, 2022 were included in the current systematic review. Among these 66 studies, the most commonly involved species was human (19 studies), followed by mouse (18 studies), swine (14 studies), rat (13 studies), calf/bovine (4 studies), sheep/lamb (4 studies) and chick (1 study). In addition, in these experiments, the most frequently utilized tissue source was adult thyroid tissue (46 studies), followed by embryonic stem cells (ESCs)/pluripotent stem cells (iPSCs) (10 studies), rat thyroid cell lines (7 studies), embryonic thyroid tissue (2 studies) and newborn or fetal thyroid tissue (2 studies). Sixty-three studies reported relevant thyroid follicular regeneration experiments *in vitro*, while 21 studies showed an *in vivo* experiment section that included transplanting engineered thyroid tissue into recipients. Together, 12 studies were carried out using 2D structures, while 50 studies constructed 3D structures.

**Conclusions:**

Each aspect of thyroid regenerative medicine was comprehensively described in this review. The recovery of optimal hormonal equilibrium by the transplantation of an engineered functional thyroid holds great therapeutic promise.

## Introduction

Hypothyroidism is a common chronic disorder of thyroid hormone deficiency (1–3). According to the results of national epidemiological surveys, it affects up to 3.7% of the population in the USA (4), 5% in Europe (5), and 14.6% in mainland China (overt hypothyroidism, 0.9%) (6). The most common cause of hypothyroidism is chronic autoimmune thyroiditis (7); however, iatrogenic causes have become increasingly frequent due to the increased use of thyroidectomy for benign and malignant thyroid lesions. If untreated, overt hypothyroidism can eventually lead to infertility, cardiovascular disease, and other serious unfavorable comorbidities (8). Although daily levothyroxine administration for hypothyroidism is generally accepted as the gold standard therapy (2), it commonly requires lifelong compliance. Due to unbalanced hormone levels of insufficient thyroxine or overreplacement, a proportion of patients suffer from persistent complaints, such as impaired neurocognitive abilities, increased body weight, recurrent fatigue, and constipation, which diminishes the quality of life, especially in patients after total thyroidectomy (9, 10). All of these beg the question of whether other alternative therapies can help to regain optimal hormonal equilibrium in hypothyroid patients.

Cell transplantation has been considered to be an alternative physiological therapy for endocrine disorders. Inspiringly, compared to other complex organs, such as the kidney and lung, the architecture of the thyroid gland, which is relatively simple due to the absence of a ductal system, can be simply regarded as a stereoscopic assembly of numerous functional units of the follicle. Therefore, the use of homologous thyrocytes and thyroid follicles opens up the unique possibility of developing a potential therapeutic substitute to compensate for hypothyroidism. Incipient experiments successfully demonstrated that isolated thyrocytes derived from different species could retain the capacity for iodide concentration and hormone synthesis *in vitro (* 11–13), and the addition of thyrotropin (thyroid stimulating hormone, TSH) to monolayer thyroid cultures resulted in a pronounced increase in thyroid hormone (12, 14, 15). Although these two-dimensional (2D) culture thyrocytes partially recapitulate the physiological function of the thyroid, reaggregation of thyrocytes into a three-dimensional (3D) follicle is a prerequisite for a normal thyroid (16). Subsequently, to obtain functional follicles, much work focusing on folliculogenesis has been carried out. One method is to isolate follicles directly from thyroid tissue (17). The utilization of mother follicles to produce descendant follicles is another option (18). Moreover, by exploring the necessary conditions or microenvironment required for folliculogenesis, most studies have attempted to imitate this microenvironment and resynthesize follicles. As expected, under proper culture conditions or when supplemented with essential stimuli, thyrocytes could reassociate in ring-like 3D follicular structures resembling cross-sections of intact thyroid tissue (17), and these follicles were capable of thyroid hormone secretion *in vitro* (19, 20). High reproducibility of folliculogenesis is a favorable beginning for thyroid regenerative medicine; however, despite their experimental utility, follicles generated from isolated mature thyrocytes or thyroid epithelial cell strains present the unavoidable problem of deficiencies in self-renewal and differentiation. Stem cells (SCs) are considered immortal, with the ability to renew themselves and differentiate into multiple lineages. Thus, SC-derived functional 3D follicles were assumed to have the capacity to reestablish optimal hormonal equilibrium in the body due to their thyroid regeneration ability. With the advent of embryonic stem cell (ESC) technologies, it is now possible to envision cellular replacement therapies for those with either congenital or postsurgical hypothyroidism who are dependent on exogenous replacement therapy (21). Moreover, following the last decade of work, organoid technology has attracted attention due to its potential contributions to basic biology and regenerative medicine and has emerged as an essential tool for thyroid reconstruction. Thyroid organoids are recognized as functional cell aggregates with near-native microanatomy that arise from self-organizing SCs (22). Currently, thyroid organoids can be derived from ESCs, induced pluripotent stem cells (iPSCs), and organ-specific adult stem cells (ASCs) (23). Embryos and fetuses serve as the source of SCs, and experiments for thyroid organoid formation utilizing ESCs and iPSCs have been performed (21, 24–27). These trends, however, were inevitably hampered by ethical and practical difficulties. Encouragingly, based on the literature review, thyroid adult stem cells were proven to exist (28–30), which reside in adult organs and could be used as an alternative to ESCs in regenerative medicine and circumvent the above ethical and practical issues (10).

While the clinical merits of exogenous therapy versus the production of endogenous thyroid hormone by a transplanted gland can be debated, it is clear that insights gained from a better understanding of thyroid regeneration or reconstruction can lead to clinical treatments for some conditions that may necessitate thyroid regeneration over pharmacologic therapy. Therefore, herein, we performed a comprehensive literature search to first illustrate the earlier findings of the morphological and functional characteristics of isolated thyrocytes based on monolayer cultures *in vitro*. Then, we focused on interpreting the process of folliculogenesis and further explored the mechanism of thyrocyte differentiation and specification from SCs. We also described the mechanisms of thyroid regeneration *in situ* after damage *via in vivo* experiments. In addition, thyroid reconstruction models and organoid models currently used were described. Finally, we discussed and highlighted the efficacy and potential tumorigenicity of thyroid organoid transplantation *in vivo.*

## Methods

### Study search strategy

According to the Preferred Reporting Items for Systematic Reviews and Meta-Analyses (PRISMA) guidelines (31), a systematic search was independently performed by 2 investigators (Chengxiang Shan and Lei Li) using electronic databases, including Medline, EBSCO, and Embase, to identify articles published before May 1st, 2022. The listing keywords were thyroid, regeneration, and/or organoid. The “related articles” function was used to broaden the search. All studies were checked for additional relevant material when appropriate. Only studies published in English were considered.

### Study selection and data extraction

Abstracts or full-text articles were initially screened and then selected or rejected by the two reviewers (Huajin Zeng and Qixuan Sheng) based on the inclusion and exclusion criteria described below. The inclusion criteria were as follows: (1) Original experiments referring to regenerative medicine of the thyroid gland; (2) Research subjects could be derived from all species (human, mouse, rat, etc.) and different sources. The exclusion criteria were as follows: (1) Review articles; (2) Nonthyroid experiments; (3) Cultures or organoid models referring to thyroid cancer; (4) Books, posters, conference abstracts, or presentations; (5) Studies with the same research subjects published repeatedly by different journals. The two reviewers (Qiang Wang and Wei Zhang) independently extracted details from each eligible study, including first author, year of publication, subject species and source, research model, key experiment process, and significant findings.

## Results

**Figure 1:**
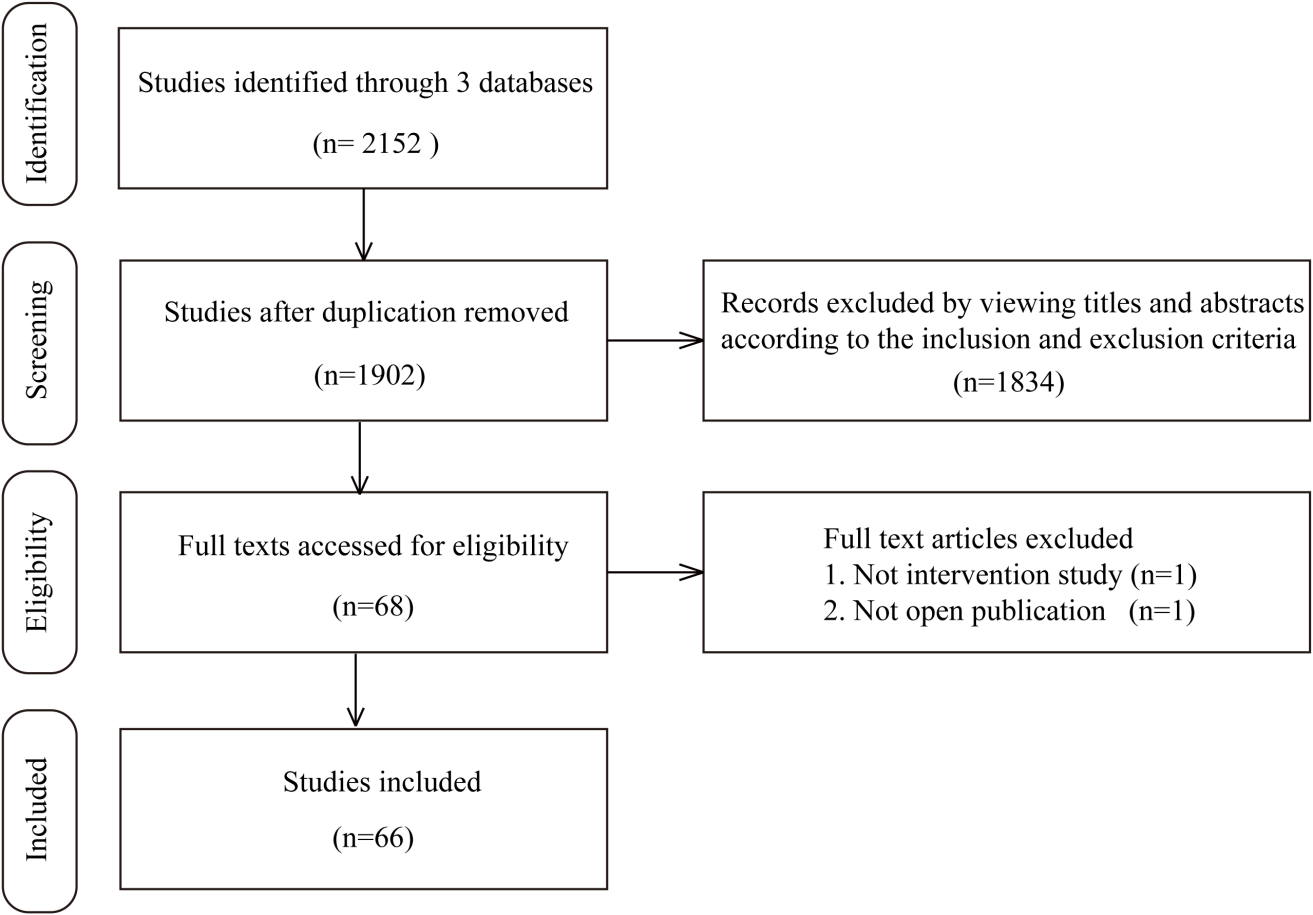
The procedures of the searching and selecting of studies included in this review.

In the study retrieval period, there were 1519 results in Medline, 332 results in EBSCO, and 301 results in Embase that matched the keywords thryroid and (regeneration or organoid). After rigorous screening based on the inclusion and exclusion criteria, we ultimately identified 66 independent studies published between 1959 and May 1st, 2022 in this systematic review. Among these 66 studies, the most commonly involved species was human (19 studies), followed by mouse (18 studies), swine (14 studies), rat (13 studies), calf/bovine (4 studies), sheep/lamb (4 studies) and chick (1 study). In addition, in these experiments, the most frequently utilized tissue source was adult thyroid tissue (46 studies), followed by ESCs/iPSCs (10 studies), rat thyroid cell lines (7 studies), embryonic thyroid tissue (2 studies), and newborn or fetal thyroid tissue (2 studies). Sixty-three studies reported relevant thyroid follicular regeneration experiments *in vitro*, while 21 studies contained an *in vivo* experiment section that included transplanting engineered thyroid tissue into recipients. Together, 12 studies were carried out based on 2D structures, while 50 studies constructed 3D structures. A flow chart illustrating the selection of studies is shown in **Figure 1**. The detailed characteristics of the included studies for thyroid regenerative medicine are summarized in **Supplemental Table 1**.

### Earlier experiments of in vitro 2D thyrocytes models

Fourteen studies between 1959 and 1978 establishing 2D thyrocyte models *in vitro* were identified. These experiments were performed with a variety of species, such as human (32, 33), calf/bovine (12, 34–36), sheep/lamb (11, 13–15), rat (37), chick (38), and swine (39, 40). The majority were carried out using 2D thyroid models, which were generated from culturing isolated thyrocytes as a monolayer. All these earlier experiments demonstrated that *in vitro* isolated thyrocytes maintained the capacity of iodide uptake, organification, and deiodination (11, 40). However, Pulvertaft R J (32) observed that a proportion of thyrocytes transformed and dedifferentiated during long-term culture (>3 weeks) and lost the capacity for iodide concentration and incorporation, which was consistent with the findings by Kalderon A E (15). Moreover, the thyrocytes’ capacity for iodide metabolism could be enhanced if additional TSH was incorporated into the culture medium, which was partially mediated *via* adenyl cyclase activation (36). However, Tong W (12) demonstrated the opposite result that extra TSH did not affect the capacity of iodide uptake. In addition to TSH, prostaglandin E 1 (PGE1) and dibutyryl cyclic AMP (DBcAMP) also enhanced iodide uptake (36) and organification (40). 7-Oxa-13-prostynoic acid inhibited the effects of TSH and PGE1 upon iodide concentration (36), and high concentrations of iodine inhibited thyrocyte organic iodination (35), as did methimazole (13, 33). It was first demonstrated by Rousset B (40) that isolated thyrocytes maintained the capacity to secrete T4, and TSH (1-60 munits/ml) could stimulate the release of T4 in a dose-related manner *in vitro*.

As no follicles were observed in the suspension (40) or monolayer culture before, three special experiments at this stage were designed to show that isolated thyrocytes were capable of self-organization and forming functional follicles. It was described that adult or embryonic thyrocytes reaggregated and reconstituted follicles when cultured in a chick chorioallantoic membrane (CAM) or rat anterior eye chamber (AEC) (37, 38). Even in monolayer culture, highly concentrated thyrocytes were rearranged into follicular structures upon TSH stimulation (39).

### Subsequent experiments focusing on thyrocyte-derived folliculogenesis

Twenty-four studies between 1979 and 2003 focusing on thyrocyte-derived folliculogenesis were identified. The most common species involved in these studies were swine (16–18, 41–48), rats (17, 49–56) and humans (17, 19, 52, 57–60). On the one hand, a proportion of experiments revealed that both directly isolated thyroid follicles (17) and newly formed follicles retained the main follicular functional characteristics, such as active iodide transport, thyroglobulin (TG) and T3 production, and TSH responsiveness (57), compared with native thyroid follicles. On the other hand, the majority of experiments focused on exploring crucial factors affecting folliculogenesis based on isolated thyrocytes or thyrocyte cell strains.

First, a high cell density was proven to be a necessity for reconstructing functional follicles in an earlier experiment (39). Its importance was reemphasized here by Fayet G, who revealed that high cell density could be achieved if the plastic medium was treated with poly-L-lysine (41). Second, appropriate cell-matrix interactions in 3D systems have been observed to enhance thyrocyte organization and function (54). When culturing thyrocytes in untreated polystyrene dishes (16), similar to suspension culture, their aggregates formed hollow spheres encompassed by a single layer of cells, similar to follicles. However, such follicular-like spheres were not identical to physiological follicles, as they possessed the opposite polarity, with the apical microvilli pointing toward the culture medium and the basal membrane pointing toward the follicular lumen. These inverted follicles (so-called inside-out follicles), which were also demonstrated by Chambard M (42) and Kitajima K (45), could regain cellular polarity and reorganize normal follicle structures when the thyrocytes were embedded in gelatin (16), collagen gel (42, 46, 57), Matrigel (19), or an alginate bead system (53). Furthermore, if dispersed thyrocytes were cultured between two layers of collagen gel, instead of mixing with the collagen gel, they could form more follicle-like structures with distinct cellular polarity (58). Third, the importance of stimulation by TSH or other specific stimuli or inhibitors should also be highlighted during follicle formation. Upon stimulation with TSH, thyrocytes aggregate and rearrange in 3D structures resembling cross-sections of intact thyroid tissue (16, 41, 43). Adding insulin to the TSH-containing culture medium stimulated the construction of follicles and the recovery of iodine metabolism of thyrocytes. Epidermal growth factor (EGF) alone could induce a progressive migration of thyrocytes radiating from the preexisting (mother) follicles into the collagen lattice and might play a role in the process of multiplication and migration of thyrocytes and formation of new follicles in the thyroid (47). Abundant oxygen was also proven to protect thyroid follicles from death due to findings in the air exposure (AE) culture system (48). Adding methimazole to the alginate bead culture system significantly decreased follicular diameters, which indicated an inhibitory activity in modulating follicular morphology (54). Fourth, the role of the inter- or intracellular signals in promoting folliculogenesis was also established. Thyrocytes cultured *in vitro* formed widely different sizes of cell colonies, indicating that the intrinsic growth rates among the same cells were heterogeneous. However, these cells did not grow individually or independently from each other; instead, they interacted with each other with synchronization. The morphological change of one single cell might initiate the interaction among neighboring cells within the aggregate, thus leading to folliculogenesis (52). Additionally, when mixing FRTL thyroid cells with dermal or thyroid fibroblasts simultaneously in matrix medium, the integrated thyroid gland equivalent (TGE) forms functional thyroid follicles (49). Nevertheless, connexin 23 (*Cx23*) overexpression in FRTL cells increased cell-to-cell communication and induced 3D follicular-like structure formation (55). The low-shear culture environment present in simulated microgravity also assisted folliculogenesis. Compared with traditional culture, both human isolated thyrocytes and FRTL-5 thyroid cells aggregated and formed densely arranged multicellular follicles enclosing a central lumen in a rotary cell culture system (56, 60); however, additional supplements with basement membrane extract and keratinocyte growth factor needed to be added simultaneously. It is worth noting that newly formed descendant follicles could also be derived from preexisting mother follicles through three types, namely, the solid nest type, the budding type and the lumen-dividing type, and the frequencies of solid nest, budding and lumen-dividing types of follicle formation were approximately 20%, 40% and 40%, respectively (18).

### Recent experiments exploring thyrocyte differentiation from SCs

Fifteen studies between 2003 and 2021 exploring thyrocyte differentiation from SCs were identified. The most common species involved in these studies were mice (9, 21, 28, 61–68) and humans (9, 21, 30, 69–71). The main purpose of these experiments was to elucidate the molecular mechanisms leading to the process of SC-derived thyrocyte specification, and these experiments were arbitrarily separated into two categories according to the source of differentiated thyrocytes, ESCs/iPSCs or ASCs/progenitor cells.

In mouse models, experiments have been conducted in which ESCs became committed to differentiating into thyrocyte-like cells *in vitro (* 61, 63, 66), and the inactivation of one allele of the TSH receptor (*TSHR*) gene in ESCs did not alter the developmental program during differentiation (62). ESCs have the potential to differentiate into the thyrocyte lineage, and some additional prerequisites might still be required to promote follicular morphogenesis. Four thyroid transcription factors (*NKX2-1*/*PAX8*/*FOXE1*/*HHEX*) were shown to be essential regulators during thyroid organogenesis (72). Coexpression of *NKX2-1* and *PAX8* in ESCs initiated a change in the fate of cells toward a thyroid follicular cell lineage in mice, and knockdown of *NKX2-1*, *PAX8* and *FOXE1* decreased the responses to TSH of thyroid-specific genes (*NIS*, *TSHR*, *TG*, and *TPO*) to different degrees (71). However, in undifferentiated ESCs, merely coexpressing *NKX2-1* and *PAX8* appeared to be insufficient for full thyrocyte fate determination. With the removal of leukemia inhibitory factor (LIF) and supplementation with activin A (endoderm induction) plus TSH (stimulus) in the culture medium, *NKX2-1*+/*PAX8*+ ESCs were capable of forming embryoid bodies (EBs) first and then transforming to mature thyroid follicular cells with as high as normal expression of thyroid-specific genes (64). This identical differentiation process was also successfully demonstrated in a double-transfected *NKX2-1*+/*PAX8*+ human ESC line (Line H9) (70) and *NKX2-1*+/*PAX8*+ iPSCs (65). Intriguingly, if the endoderm-induction step (exclusion of activin A) in the above multistage differentiation protocol was omitted, *NKX2-1*+/*PAX8*+ ESCs differentiated into nonthyrocyte lineages and mature thyrocytes, suggesting that additional factors specifically restricted the progenitors to the thyroid lineage (68). In addition, bone morphogenetic protein (BMP) and fibroblast growth factor (FGF) signaling pathways were found to be active in *NKX2-1*+ endodermal progenitors *in vitro*, and endogenous *NKX2-1* expression could also be directly induced by culturing ESCs/iPSCs in medium supplemented with BMP4 and FGF2 (21). Furthermore, transforming growth factor-β (TGF-β) signaling was also demonstrated to be significant during the transition from the immature to mature thyrocyte state, increasing the capacity for iodide uptake and organification (68).

There was sufficient evidence that tissue-specific stem cells resided in certain adult tissues, as was demonstrated in the thyroid. Side population (SP) cells, recognized by the ability to efflux the vital dye Hoechst 33342, are generally considered to have characteristics consistent with stem cells. A cell population with the SP phenotype was proven to exist in both mouse thyroid and human thyroid. SP cells appeared in the interfollicular space but not in follicles. They represented 0.3% to 1.4% of the total population of cells in mouse thyroid (28) and 0.1% in human thyroid (30), which were eliminated by verapamil pretreatment. The positive expression of *SCA1*, *CD34*, *Nestin*, *OCT4*, and *Nanog*, as suggested stemness markers, was found in a proportion of putative thyroid SCs (69), while in some cases, emblematical stemness markers were not easily identified. It was known that isolated thyroid stem cells could maintain viability with phenotype during passaging *in vitro*, but they showed resistance to growth stimulations and were unable to differentiate directly, which was perhaps due to lack of interaction between stem cells and niche cells. One possible option to induce murine thyroid ASC differentiation into functional follicular cells is to generate thyrospheres or thyrospheroids containing ASCs in specific “spheroid medium” primarily supplemented with EGF and bFGF. For human thyrosphere formation, additional WNT and R-Spondin1 were required in the defined thyroid gland medium (9). Once thyrospheres are established and then cultured in the “differentiation medium”, they are specified into mature thyroid cells expressing thyroid-specific genes, producing thyroid hormones (69), and showing TSH-dependent I^125^ uptake (30).

### Current thyroid reconstruction models and thyroid organoid models

Twenty-two studies between 1984 and 2022 exploring thyroid reconstruction models and thyroid organoid models were identified. The most common species involved in these studies were mouse (9, 21, 25, 63, 65–68, 73, 74), human (9, 19, 21, 26, 52, 59, 60, 69, 74, 75), rat (49, 52, 54, 56, 75, 76) and swine (77). Detailed 3D matrices and medium components for different thyroid cell types were concluded in **Table 1**.

**Table 1:** Detailed 3D matrices and medium components for different thyroid cell types applied in researches.

Researcher year(Ref)	Species	Thyroid cell types	3D matrices	Medium components
Bell E 1984(49)	Rat	FRTL cells	Rat tail tendon collagen	Each dish contained 2.3 ml 1.76×DMEM, 30μL/100-fold concentrate of six hormones, 0.25 ml 0.1 N NaOH, 0.22 ml FBS, 1.5 ml 1.6 mg/ml rat tail tendon collagen, and 2.0×10^5^ rat skin or thyroid fibroblasts and 1.5×l0^5^ thyroid epithelial cells suspended in 0.75 ml DMEM-10% FBS.
Nitsch L 1984(50)	Rat	T78 cells	Agarose	Coon’s modified Ham’s F12 medium containing 0.5 % calf serum and six hormones, and growth factors, and 10 mU/ml bovine TSH.
Mulcahy R T 1985(51)	Rat	FRTL-5 cells	0.5% agar	Modified Ham's F12 medium supplemented with 5% calf serum and five hormones. [TSH (0 to 10 mU/ml); tydrocortisone (10 nM), insulin (10 mg/ml), transferrin (5 mg/ml), and glycyl-Lhistidine-L-lysine acetate (10 ng/ml).]
Derwahl M 1990(52)	Rat	FRTL-5 cells	Collagen gel	Coon's modified Ham F12 medium supplemented with 5% calf serum and six hormones. [glycyl-histidyl-lysine, 10 ng/ml; insulin, 10 mg/ml; somatostatin, 10 ng/ml; transferrin, 5 mg/ml; hydrocortisone, 3.2 ng/ml; bovine TSH, 10 mU/ml, 100 IU penicillin/ml; and 100 ng streptomycin/ml.]
Bürgi U 1998(53)	Rat	FRTL-5 cells	Alginate beads	N/A
Tonoli H 2000(55)	Rat	FRTL cells	N/A	Coon’s modified Ham’s F-12 medium containing 100 U/ml penicillin and 0.1 mg/ml streptomycin and supplemented with 5% FCS and a four-hormone mixture. [10 mg/ml insulin, 10 nm hydrocortisone, 5 mg/ml transferrin, and 10 ng/ml glycyl-l-histidyl-l-lysine acetate.]
Green L M 2002(56)	Rat	FRTL-5 cells	Bioreactor vessels	DMEM/F12 medium (50:50 v/v) supplemented with 5% calf serum, 2 mM glutamine and a six-hormone mix. [10 ng/ml somatostatin, 10 ng/ml glycyl-l-histidyl-l-lysine acetate, 5 μg/ml transferrin, 10 nM hydrocortisone, 1×10^-3^ U of TSH, and 10 μg/ml insulin.]
Lin R Y 2003(61)	Mouse	ESCs	0.1% gelatin.	DMEM supplemented with 15% FCS, penicillin-streptomycin (100 U/ml), 1% supernatant LIF, and 1.5×10^-4^ m monothioglycerol. EBDM: IMDM supplemented with 15% FCS, 0.5 mg/ml ascorbic acid, and 1.5×10^−4^M monothioglycerol.
Arufe M C 2006(62)	Mouse	ESCs	0.1% gelatin	DMEM supplemented with 15% FCS, penicillin-streptomycin (100 U/ml), 10 ng/ml LIF, and 1.5×10^-4^ M monothioglycerol. EBDM: IMDM supplemented with penicillin/streptomycin, 15% FCS, 2 mM L-glutamine, 5% protein-free hybridoma medium (PFHM-II), 0.5 mM ascorbic acid, transferrin (200 μg/ml), and 1.5×10^−4^ MMTG.
Antonica F 2012(63)	Mouse	ESCs	GFR-Matrigel	DMEM supplemented with 15% ES-certified FBS, 0.1 mM non-essential amino acids. 1 mM sodium pyruvate, 0.1 mM 2-mercaptoethanol, 50 U/ml penicillin/50 µg /ml streptomycin and 1,000 U/ml LIF. EBDM: The above medium without LIF, supplemented with 50 µg/ml ascorbic acid but supplemented with 1 µg/ml doxycycline and 1 mU/ml hTSH when indicated.
Ma R 2013(64)	Mouse	ESCs	Gelatin	DMEM supplemented with 15% FCS, penicillin-streptomycin (100 U/mL), 1.5×10^−4^ monothioglycerol, and 10 ng/mL LIF. EBDM: DMEM supplemented with penicillin/streptomycin, 15% KnockOut serum replacement medium, 5% protein-free hybridoma medium (PFHM-II), 1.5×10^−4^ M monothioglycerol, and 1000 μU/mL human recombinant TSH.
Kurmann A A 2015(21)	Mouse	ESCs iPSCs Fibroblasts	Pure growth factor reduced Matrigel	Complete serum-free differentiation medium supplemented with 250 ng/ml mFGF2, 100 ng/ml hFGF10, 50 ng/ml mIGF-I, 25 ng/ml hEGF, 100 ng/ml Heparin Sodium Salt, 10 µg/ml insulin, and 1 mU/ml bovine TSH. EBDM: Complete serum-free differentiation medium consisting of 75% IMDM and 25% Ham’s Modified F12 medium supplemented with N2 and B27+RA, 0.05% BSA, 200 mML-glutamine, 0.05 mg/ml ascorbic acid and 4.5×10^-4^ M monothioglycerol
Ma R 2015(70)	Human	ESCs (Line H9)	Gelatin/Matrigel	Feeder-free culture conditions with mTeSR medium.
Ma R 2015(65)	Mouse	iPSCs	Growth factor-restricted Matrigel	DMEM supplemented with 15% FBS, l-glutamine, penicillin/streptomycin, non-essential amino acids, β-mercaptoethanol, and 1,000 U/ml LIF.
Antonica F 2017(65)	Mouse	ESCs	Matrigel	Mouse ESCs are routinely cultured in DMEM, 15% FBS (ES qualified FBS), supplemented with LIF (1000 U/mL final), nonessential amino acids/ml (0.1 mM final), sodium pyruvate (1 mM final), penicillin and streptomycin (50 U/mL final), and 2-mercaptoethanol (0.1 mM final). EBDM: DMEM supplemented with 15% FBS, vitamin C (50 μg/mL final), nonessential amino acids (0.1 mM final), sodium pyruvate (1 mM final), penicillin and streptomycin (50 U/mL final), and 2-mercaptoethanol (0.1 mM final).
Pan J 2019(75)	Rat	FRTL-5 cells	Decellularized thyroid	Coon's modified Ham's F-12 medium supplemented with 5% bovine serum and a six-hormone mixture. [bovine TSH (1 mU/Ml), insulin (10 μg/mL), hydrocortisone (0.35 ng/mL), transferrin (5 μg/mL), glycyl-L-histidyl-L-lysine acetate (2 ng/mL), and somatostatin (10 ng/mL).]
Romitti M 2021(68)	Mouse	ESCs	Growth- factor-restricted Matrigel	Mouse ESCs were cultured and were differentiated as described previously by Antonica F, 2012.

3D, Three-dimensional; FBS: DMEM, Dulbecco's modified eagle medium; FCS, Fetal calf serum; LIF, Leukemia inhibitory factor; TSH, Thyrotrophin, Thyroid Stimulating Hormone; ESCs, Embryonic stem cells; iPSCs, Induced pluripotent stem cells; mFGF, Recombinant mouse fibroblast growth factor; hFGF, Recombinant human fibroblast growth factor; mIGF-I, Insulin-like growth factor-I; hEGF, Recombinant human epidermal growth factor; ES, Embryonic stem cell; EBDM, Embryoid bodies differentiation medium; IMDM, Iscove’s modified Dulbecco’s medium.

As described above, differentiated thyrocyte lineages have been successfully induced from ESCs/iPSCs or ASCs/progenitor cells. Consequently, thyroid reconstruction could originate directly from these SCs (9). In a mouse model, *NKX2-1*+/*PAX8*+ ESC-derived thyroid cells organized into 3D follicular structures and maintained thyroid functionalities (63, 66, 68). The culture of *NKX2-1*+ ESCs/iPSCs in 3D Matrigel conditions triggered the formation of follicular-like clusters of cells (21, 65). Furthermore, in *FGF10 EX1* ^mut^/*EX3* ^mut^ transgenic mice with severe thyroid hypoplasia, allogeneic normal ECSs could induce orthotopic thyroid tissue regeneration when microinjected into the embryos of *FGF10 EX1* ^mut^/*EX3* ^mut^ mice *via* blastocyst complementation (25). Thyroid organoids can also be generated *in vitro* by culturing isolated adult or fetal thyroid cells in a specific medium supplemented with a necessary stimulus (such as EGF, FGF10, WNT, R-Spondin1, or Noggin) (26, 74). Human ASC-derived thyrospheres seeded in collagen gel in the presence of a “differentiation medium” started to generate functional thyroid follicles (9, 69). In a mouse model, isolated cells formed thyroid organoids when embedded into an organoid culture medium containing Matrigel and various growth factors, and each thyroid organoid appeared to be composed of clonally derived cells and did not simply represent cellular aggregates (67). Interestingly, some authors generated small or microthyroid tissues by directly culturing mature thyrocytes or thyroid cell lines in a 3D medium (19, 49, 52, 54, 56, 60) and proved their physiological functions with enriched critical genes (20, 59). However, their organoids’ capabilities of self-renewal and differentiation did not seem to be fully determined.

Some special protocols for constructing thyroid organoids have also been reported. Arauchi A (76) showed thyroid reconstruction using the cell sheet technique. After transplantation into thyroidectomy-inducible hypothyroid rats, these cell sheets were organized into honeycomb-like structures of thyroid follicles. Moreover, parafollicular cells and numerous functional microvessels containing red blood cells also existed among the follicles, telling the reliable capability of cell sheet technique to get new blood supply. Bulanova E A (73) described thyroid reconstruction from embryonic thyroid tissue-derived spheroids (TS) and embryonic allantoic tissue-derived spheroids (AS) using a multifunctional Fabion 3D bioprinter with the turnstile system. Fusion spheroids with three TS and six AS formed the rudiment of thyroid organoids, and during further culture in collagen gel, the endothelial progenitors in AS responded to the angiogenic factor secreted by TS and then initiated vascularization in bioprinted thyroid. Yang Y (77) accomplished thyroid organoid formation by microencapsulation of porcine thyroid cells by applying a designed 8-nozzle microfluidic device. Upon TSH stimulation, thyroid cells gathered and formed 3D follicles in the inner core of microbeads within 48 hours and produced more thyroxine than monolayer cultured thyrocytes. Pan J (75) also employed a decellularization-recellularization method to reconstruct multifunctional organoids consisting of both thyroid cells and parathyroid cells. A rat acellular thyroid scaffold with an intact follicular basement membrane and arterial elastic fiber network was first obtained by thyroid artery perfusion with sodium dodecyl sulfate. Alternative decellularization protocols were also provided by Alfieri M (78). Upon the subsequent coseeding of human thyroid cells and parathyroid cells into this rat decellularized matrix, all of these cells were capable of distributing throughout the scaffold and expressing critical proteins, such as TG, TPO, and parathyroid hormone (PTH).

### *In vivo* experiments of thyroid regeneration after thyroidectomy or damage

Four studies between 2012 and 2021 focusing on *in situ* compensative thyroid regeneration after thyroid damage were identified (79–82), and all of them were performed in mice. It was demonstrated that the central part of the intact thyroid lobe where many small or micro follicles were present generally served as a center for proliferation, where newly formed follicles were generated and pushed to the peripheral zone during maturation. When partial thyroidectomy was performed, on the one hand, the primitive thyroid follicles in the center of proliferation underwent a regeneration process (the budding type of folliculogenesis) to produce new follicles (81). On the other hand, the proliferative center extended to the area near the cut edge, and some follicles in the area became irregular in shape, and this affected area was considered under repair after damage. In the proliferative area, some class of immature clear cells was found to participate in the repair or regeneration of the thyroid gland (79); however, the nature and origin of these clear cells and their relationship to follicular cells or parafollicular cells (C cells) were not fully elucidated. Several crucial stemness markers were monitored to demonstrate whether stem cells or progenitor cells played a part in the process of thyroid regeneration after thyroid damage. In a transgenic *TPOCreER2*/*iDTR* mouse model, after 4 weeks of combined administration of tamoxifen (TM) and diphtheria toxin (DT), more than 90% of *TPO*-expressing mature thyroid follicles were damaged in mice. A significant increase in stem cell markers (*OCT4*, *Nanog*, *SOX* and *REX1*) during and following TM/DT treatment with a transient surge at 2 weeks was intuitively displayed (82). Furthermore, stem cell antigen 1 (*SCA1*)-positive cells were initially found to have negative *NKX2-1* expression in nonfollicular mesenchymal areas, suggesting that *SCA1*+ cells were not of thyroid follicular cell origin. However, at 120 days after surgery, these *SCA1*+ cells subsequently differentiated to *SCA1*+/*NKX2-1*+ cells, indicating that *SCA1*+ cells might exhibit stem-like or progenitor-like cell characteristics and be responsible for the regeneration of thyroid follicular cells after surgery (80). Keratin14 (*KRT14*), known as a liver progenitor marker, was also identified in a limited part of the proliferative area. However, *KRT14*+ cells were demonstrated to have no specificity, as they could be found in both follicular cells and nonfollicular areas (80).

Concerning thyroid function recovery after partial thyroidectomy or damage, in most conditions, serum FT4 or T4 levels could return to normal at 2 to 8 weeks after damage, but TSH levels were still elevated with statistical significance (81).

### The efficacy and potential tumorigenicity of *in vivo* transplantation

Eleven studies between 1984 and 2022 investigating the efficacy and potential tumorigenicity of thyroid grafts were identified. Eight of them elucidated the efficacy of artificial thyroid gland transplantation, mainly into hypothyroid animals, and 3 of them specifically displayed the potential tumorigenicity of the implanted grafts.

To assess regenerative efficiency and function, the explanted thyroid tissues were routinely examined by histology, immunodetection, protein, or gene expression assays (83). First, the morphological formation of thyroid-like tissue in host animals was shown after transplantation. It was found that no matter which kinds of thyroid organoids were implanted under the skin or beneath the kidney capsule, one or two months later, the transplanted grafts exhibited numerous follicles formed by monolayered epithelium at the grafting sites (9, 19, 21, 26, 49, 59, 63, 65, 73), verified by cytosolic TG expression and TG deposition within the follicular lumens. Second, the harmonic function of implanted thyroid organoids at different quantities was illustrated. In totally thyroidectomized rats, the experimental group with 1×10^6^ cells of TGE grafts showed a significant increase in body weight, although the final weight attained fell short of that of the control group with intact thyroid glands (49). Additionally, transplanting thyroid cell sheets with 2.5×10^6^ cells into rats following thyroidectomy significantly elevated both FT3 and FT4 at 2 weeks after surgery, while transplanting half the number only contributed to FT4 recovering significantly at 3 weeks and to less than the normal level when FT3 and FT4 levels were saturated at 4 weeks (76). It was noticeable that in nonthyroidectomized rats, the TGE graft was not able to form follicles or change their weights (49). Similarly, in euthyroid severe combined immunodeficient (SCID) mice, no matter how many thyrocytes of the human thyroid-derived organoid were transplanted, the total T4 level in recipient mice did not differ significantly from that in normal SCID mice (19, 59). An insufficient TSH feedback loop stimulation induced by normal TSH levels in euthyroid animals may account for these phenomena. In I^131^ inducible hypothyroid mouse models, 3 and 5 weeks after grafting three TS and six AS, recipient mice showed a gradual normalization of body temperature and a substantial elevation of serum T4 levels, while T4 levels were still far from the normal range (73). After transplanting 2.5-3×10^6^ cells from *NKX2-1*+/*PAX8*+ ESC-derived organoids beneath the unilateral kidney capsule, hypothyroid mice showed a full normalization of plasma thyroid hormone levels (21) and body temperature (63) 4 or 8 weeks after grafting. Reconstitution of T4 levels was accompanied by amelioration of elevated plasma TSH levels, further emphasizing that physiologic rescue was occurring (21). However, when injecting 6×10^5^ cells from adult thyroid-derived organoids underneath the kidney capsule, although the area of the follicular structure remained stable at the grafting site, the FT4 levels only increased very modestly (9).

Regarding the tumorigenicity of thyroid organoids, no reports of tumor events were noted after *in vivo* transplantation in all of the recipients during their research periods. No tumor development was observed even in xenografted SCID mice (69), nor was it observed when transplanted *p53* knockout thyroid-derived organoids were subcutaneously injected into BALB/c nu/nu mice (67). In addition, no macroscopic tumors were detected at 1 year after transplantation within the thyroid organoid implants that were previously exposed to X-ray radiation before transplantation (9). Intriguingly, gene expression analysis showed that more than 70% of upregulated genes related to thyroid carcinogenesis were downregulated in early passage thyroid organoids and continued to be downregulated in later passage thyroid organoids. Correspondingly, more than 70% of downregulated genes related to thyroid carcinogenesis were upregulated and continued to be upregulated by prolonged culture. These data indicated that prolonged culturing did not result in the activation or inhibition of tumor-related markers.

## Discussion

Hypothyroidism is one of the most common hormone deficiency problems. Although hormone supplemental therapy can be easily performed by daily levothyroxine administration, a constant exogenous thyroid hormone dosage does not meet the requirements associated with growth, puberty, pregnancy, and stress, thus leaving room for new therapeutic approaches (84). The applications of tissue engineering and regenerative medicine for thyroid hormone deficiency management are highly desirable (85). To verify whether regenerated thyroid cells, tissues, or organs would accommodate the variation in hormone demand, we undertook this systematic review. We intended to present the currently applied regenerative thyroid models and highlight their efficacy and potential tumorigenicity *in vitro* and *in vivo*, while it was arduous to interpret the entire framework without understanding the characteristics of thyrocytes and thyrocyte-derived follicles and the process and mechanism of folliculogenesis and lineage differentiation, which were absent in previous reviews. Besides, few up-to-date published reviews conclude the exploration to the development of thyroid regenerative therapy from temporal, technical and conceptual perspectives comprehensively. Therefore, various aspects concerning thyroid regenerative medicine were exhibited in detail here, but there are still some debatable issues that need to be outlined and further discussed.

To begin with, in the earlier experiments, discrepancies existed among the metabolic properties of isolated thyrocytes prepared in various laboratories. Differences in experimental techniques might be part of the reason for this. Another explanation is the thyrocytes isolation procedure used during this earlier stage of experimentation, which was always accomplished by trypsinization that could cause reversible membrane injuries and leach important cofactors from the cells, thus changing the responsiveness of the thyrocytes. Additionally, freshly trypsinized cells are sensitive to mechanical injuries.

Next, different experimenters independently reported that inverted follicles or noninverted follicles were observed in suspension culture (42, 50) and that the polarity of follicles was retained in a medium containing 0.5% fetal calf serum and reversed in a medium containing 5-10% serum (86). Thus, the process of follicle polarity reversal should be emphasized. It was likely that the migration of tight junction elements occurred first in polarity reversal. These sealing-strand elements, originally located at the luminal margin of the lateral plasma membrane, came to occupy the whole lateral plasma membrane and then appeared at the end facing the culture medium. Therefore, the interaction of cell membranes with extracellular components with adhesive properties appeared to be a determinant factor in the orientation and stabilization of follicle polarity (45). In addition, newly synthesized basal lamina might come to occupy the luminal surface because the osmotic pressure was weaker here than in the culture medium.

Another problem was that although a proportion of included researchers applied the term “organoid” in their experiments and demonstrated maintained morphological integrity, functional activity and ability to proliferate *in vitro*, the thyroid reconstruction models they built did not strictly conform to the concept of organoids. Formerly, organoids were defined as scaled-down, miniature versions of an organ generated *in vitro* in a 3D culture system that can recapitulate their micro environment *in vivo*. However, recently, the definition of organoids has been narrowed down to self-organizing 3D structures grown from SCs that mimic the *in vivo* environment, architecture and multilineage differentiation of the original organs (87, 88). Therefore, the term reconstructed thyroid or regenerated thyroid might be more appropriate in defining non-SC-derived thyroid organoids.

Furthermore, most thyrocytes in the thyroid appeared to be able to respond by a few divisions when encountering damage, despite the known fact that the thyroid is a special endocrine organ with very slow turnover. Once differentiation is completed, the tissue grows roughly in parallel with body weight and remains at the same size throughout adult life. The proliferative cells in the thyroid were reminiscent of thyroid ASCs, whereas thyroid stem-like cells have been discussed for decades without a general consensus (89). Although SP cells, *SCA1*+ cells and other types of cells with positive stemness markers were found in the thyroid and served as ASCs, in some cases, emblematical stemness markers were clearly absent. van der Vaart J (74) indicated that thyroid tissue might not rely on a “professional” stem cell for their maintenance and repair but rather temporarily recruit differentiated cells into a transient progenitor pool, similar to the liver (90). Another assumption for distinctive growth rates of folliculogenesis was the heterogeneity of ASCs in terms of cell cycle state, which might be composed of both quiescent (out of cell cycle and in a lower metabolic state) and active (in cell cycle and not able to retain DNA labels) stem cell subpopulations. Quiescent stem cells experience a diverse duration of refractory time before entering into active stem cells, which has been demonstrated in hair follicle stem cells (91). Here, we believe that thyroid ASCs exist but still raise the question of whether ASCs express undiscovered or unknown stemness markers.

The efficacy of thyroid organoid transplantation *in vivo* should be emphasized once again. We found an elevation in serum T4 levels after thyroid graft transplantation into hypothyroid animals, which indicated that these grafts accommodated the hormone demand to a certain degree. Although the increases in thyroid hormone levels were not proportional to the implanting cell numbers (76), the amplitude of hormone increase seemed directly correlated with the cell amount, and a functional endocrine substitute would have an extreme reliance on the injection of a sufficient number of cells. In addition, the number of blood vessels that grew into the xenograft was also crucial. The nourishment and oxygen from the newly developed vessels promoted the generation of thyroid tissue-like follicle structure.

Despite all the progress made in the development of thyroid organoids *in vitro* and animal models, some crucial issues have to be addressed when it comes to clinical application. To avoid the potential immune response of graft, autologous transplantation is encouraged. While for thyroidectomy patients with thyroid carcinoma who account for a large proportion of patients suffering from hypothyroidism, the potential tumorigenicity of autologous thyroid cells is nonnegligible. A long-term observation of tumorigenicity lasting for decades in thyroid transplants is absent *in vivo* experiments, which challenges the real-world application and requires more evidence for safety evaluation. What’s more, TSH inhibition therapy is essential for the post-surgery management of thyroid carcinoma. The time of transplantation and the number of thyroid organoid grafts need to be cautiously and exactly controlled and calculated. These all call for further well-designed studies.

## Conclusions

In the field of thyroid regeneration medicine, significant achievements and advances in the last three decades have been presented in detail in this review. With the development of stem cell technology and organoid technology, recovery of thyroid hormone levels by transplantation of reconstructed thyroid derived from stem cells *in vitro* holds great therapeutic promise.

## Data availability statement

The original contributions presented in the study are included in the article/**Supplementary Material**. Further inquiries can be directed to the corresponding authors.

## Author contributions

Research design: CS; Systematic search and study selection: LL, QS, and HZ; Initial manuscript writing: CS, LL, and QW; Manuscript revision: GM, MQ, and WL; Final approval of manuscript: CS and WZ. All authors contributed to the article and approved the submitted version.

## Funding

This work was supported by the Pyramid Talented Personnel Project of Changzheng Hospital awarded to CS.

## Conflict of interest

The authors declare that the research was conducted in the absence of any commercial or financial relationships that could be construed as a potential conflict of interest.

## Publisher’s note

All claims expressed in this article are solely those of the authors and do not necessarily represent those of their affiliated organizations, or those of the publisher, the editors and the reviewers. Any product that may be evaluated in this article, or claim that may be made by its manufacturer, is not guaranteed or endorsed by the publisher.
