# Supplementary material for: Engineering a functional thyroid as a potential therapeutic substitute for hypothyroidism treatment: A systematic review
Source: Front Endocrinol (Lausanne). 2022 Dec 2;13:1065410. doi: 10.3389/fendo.2022.1065410 (PMC9755335; doi:10.3389/fendo.2022.1065410)
Supplement: Supplementary file 1 [file Table_1.docx]

Supplemental table 1. Detailed characteristics of the included studies for thyroid regenerative medicine

| Author | Year | Species | Source | Research Model | Key Experiment Process | Significant Findings |
| --- | --- | --- | --- | --- | --- | --- |
| Pulvertaft R J^1^ | 1959 | Human | Thyroid tissue | 2D monolayer model  *in vitro* model | Thyrocytes isolation  Thyrocytes culture  NaI^131^ incorporation into medium  Microscopic examination  Radioactivity measurement  Chromatographic analysis | A proportion of thyrocytes could be transformed and became dedifferentiated after long time (>3 weeks) culture.  Untransformed thyrocytes preserved the capacity of iodide uptake.  Untransformed thyrocytes preserved the capacity of iodide organification, mainly into MIT.  TSH had no effect on the morphology and multiplication rates of thyrocytes. |
| Pastan I^2^ | 1961 | Calf | Thyroid tissue | 2D monolayer model  *in vitro* model | Thyrocytes isolation  Thyrocytes culture  NaI^131^ incorporation into medium  Radioactivity measurement  Chromatographic analysis | Isolated thyrocytes preserved the capacity of iodide uptake.  Isolated thyrocytes preserved the capacity of iodide organification, mainly into MIT.  TSH enhanced the capacity of iodide uptake.  TSH increased the capacity of I^131^ incorporation into MIT. |
| Tong W^3^ | 1962 | Sheep | Thyroid tissue | 2D monolayer model  *in vitro* model | Thyrocytes isolation  Thyrocytes culture  I^131^-labeled substrates incubation  Microscopic examination  Radioactivity measurement  Chromatographic analysis | Isolated thyrocytes preserved the capacity of iodide uptake.  Isolated thyrocytes preserved the capacity of iodide organification, mainly into MIT.  Isolated thyrocytes preserved the capacity of deiodination of iodotyrosines. |
| Hung W^4^ | 1964 | Human | Thyroid tissue | 2D monolayer model  *in vitro* model | Thyrocytes isolation  Thyrocytes culture  I^131^-labeled substrates incubation  Radioactivity measurement  Chromatographic analysis | Isolated thyrocytes preserved the capacity of iodide uptake.  Isolated thyrocytes preserved the capacity of iodide organification.  Methimazole had no effect on the capacity of iodide uptake, but did inhibit the capacity of iodide organification. |
| Kerkof P R^5^ | 1964 | Sheep | Thyroid tissue | 2D monolayer model  *in vitro* model | Thyrocytes isolation  Thyrocytes culture  I^131^-labeled substrates incubation  Radioactivity measurement  Chromatographic analysis | Isolated thyrocytes preserved the capacity of iodide uptake.  Isolated thyrocytes preserved the capacity of iodide organification into MIT, DIT, and T4.  TSH enhanced the capacity of iodide uptake.  TSH increased the iodide incorporation into MIT, DIT and T4. |
| Tong W^6^ | 1964 | Bovine | Thyroid tissue | 2D monolayer model  *in vitro* model | Thyrocytes isolation  Thyrocytes culture  I^131^-labeled substrates incubation  Radioactivity measurement  Chromatographic analysis | Isolated thyrocytes preserved the capacity of iodide organification into MIT, DIT, and T4.  TSH increased iodide incorporation into MIT, DIT, and T4.  TSH had no effect on the capacity of iodide uptake. |
| Mallette J M^7^ | 1966 | Rat | Thyroid tissue | 3D model  *in vivo* model | Thyrocytes isolation  Thyrocytes and tissue culture *in vitro*  Grafts transplantation into CAM of chick  Grafts transplantation into AEC of rat  Microscopic examination  Histochemical observation | Isolated thyrocytes had the capacity of self-organization and adhering to each other.  Isolated thyrocytes possessed the capacity to reconstitute thyroid follicles. |
| Shimoda S I^8^ | 1966 | Bovine | Thyroid tissue | 2D model  *in vitro* model | Thyrocytes isolation  Thyrocytes culture  Incubation with I^131^ and I^127^  Radioactivity measurement  Chromatographic analysis | High concentrations of inorganic iodide inhibited iodide organification in isolated thyrocytes. |
| Kalderon A E^9^ | 1967 | Lamb | Thyroid tissue | 2D monolayer model  *in vitro* model | Thyrocytes isolation  Thyrocytes culture  Histochemical observation | Aged thyrocytes did not form compact monolayers, but isolated clusters during subculture.  Cell transformation occurred in aged thyrocytes (>20 days culture), accompanied with cell structure change and loss of contact inhibition.  Additional TSH induced irregularly distributed follicle-like spaces approximately similar to their histologic counterparts.  Additional TSH increased enzymatic reaction products in younger thyrocytes, whereas in aged thyrocytes the response to TSH was reduced or lost. |
| Rodesch F^10^ | 1967 | Sheep | Thyroid tissue | 2D model  *in vitro* model | Thyrocytes isolation  Incubation with I^131^ and I^125^  Radioactivity measurement  Chromatographic analysis  Oxygen uptake analysis | Among the media used for the incubation of the cells, Earle’s salt solution was most favorable.  Isolated thyrocytes preserved the capacity of iodide uptake.  Isolated thyrocytes preserved the capacity of iodide organification into MIT, DIT, and T4.  Methimazole inhibited the capacity of iodide organification.  Mitochondria of isolated thyrocytes exhibited a satisfactory respiratory control. |
| Hilfer S R^11^ | 1968 | Chick | Embryonic thyroid tissue | 3D model  *in vitro* model  *in vivo* model | Thyrocytes isolation  Grafts transplantation into CAM of chick  Electron microscopic examination | Isolated embryonic thyrocytes possessed the capacity to reconstitute thyroid follicles.  Thyrocytes went through a lot of changes with respect to cytoarchitecture after the process of reaggregation. |
| Burke G^12^ | 1971 | Bovine | Thyroid tissue | 2D model  *in vitro* model | Thyrocytes isolation  I^131^-labeled substrates incubation  Radioactivity measurement  Chromatographic analysis | TSH, PGE1 and DBcAMP enhanced the capacity of iodide uptake in isolated thyrocytes.  PY1 inhibited the effects of TSH and PGE1 on iodide uptake of isolated thyrocytes.  TSH effects on iodide uptake were mediated via adenyl cyclase activation. |
| Fayet G^13^ | 1971 | Swine | Thyroid tissue | 3D model  *in vitro* model | Thyrocytes isolation  Thyrocytes culture  Additional TSH stimulation  Electron microscopic examination | TSH induced follicle formation from isolated thyrocytes.  Thyrocytes cultured as monolayer were poorer in organelles than reassociated cells, which meant TSH induced thyrocytes differentiation. |
| Rousset B^14^ | 1976 | Swine | Thyroid tissue | 2D model  *in vitro* model | Thyrocytes isolation  I^131^-labeled substrates incubation  Microscopic examination  Radioactivity measurement  Chromatographic analysis | No follicles were observed in the thyrocytes suspension.  Isolated thyrocytes preserved the capacity of iodide uptake, and the capacity was enhanced with additional TSH.  TSH and DBcAMP enhanced the capacity of iodide organification.  Isolated thyrocytes preserved the capacity of secreting T4.  TSH (1-60 mU/ml) stimulated the release of T4 from isolated thyrocytes in a dose-related manner.  Isolated thyrocytes preserved the capacity of deiodination of iodotyrosines. |
| Mauchamp J^15^ | 1979 | Swine | Thyroid tissue | 3D model  *in vitro* model | Thyrocytes isolation  Thyrocytes culture *in vitro*  Change the culture conditions  Electron microscopic examination | Isolated thyrocytes formed monolayers on pretreated plastic substratum.  Isolated thyrocytes formed inverted follicles with opposite polarities in untreated polystyrene dishes.  TSH, DBcAMP, PGE1, and PGE2 induced normal follicles formation in untreated polystyrene dishes.  Isolated thyrocytes formed normal follicles if gelatin was added to the culture medium. |
| Denef J F^16^ | 1980 | Rat  Swine  Human | Thyroid tissue | 3D model  *in vitro* model | Thyroid follicles isolation  Microscopic examination  Cellular viability analysis  I^125^-labeled substrates incorporation  C^14^-labeled protein analysis | A new isolation procedure for direct thyroid follicles instead of dispersed thyroid cells was reported.  Isolated follicles preserved the capacity of iodide uptake.  Isolated follicles preserved the capacity of thyroglobulin synthesis.  TSH enhanced the process of protein synthesis.  Isolated follicles preserved the main morphological and functional characteristics derived from thyroid. |
| Fayet G^17^ | 1980 | Swine | Thyroid tissue | 3D model  *in vitro* model | Thyrocytes isolation  Thyrocytes culture and subculture  Additional TSH stimulation  I^125^-incorporation into medium  Radioactivity measurement  Microscopic examination | TSH induced normal follicles formation in isolated thyrocytes suspension.  Iodide active transportation occurred in follicle, while passive diffusion in monolayer.  Newly formed follicles preserved the main morphological and functional characteristics derived from thyroid. |
| Chambard M^18^ | 1981 | Swine | Thyroid tissue | 3D model  *in vitro* model | Thyrocytes isolation  Thyrocytes culture on/in the collagen gel  Electron microscopic examination | Isolated thyrocytes formed monolayers on the collagen gel surface.  Isolated thyrocytes formed inverted follicles with opposite polarities in suspending culture.  Isolated thyrocytes formed follicles in the collagen gel.  Monolayer thyrocytes on collagen gel transformed into follicles when covering their apical surface with another layer of collagen gel.  Collagen influenced the polarized organization and stability of thyrocytes. |
| Hovsépian S^19^ | 1982 | Swine | Thyroid tissue | 3D model  *in vitro* model | Thyrocytes isolation  Thyrocytes culture and subculture  Additional TSH stimulation  Immunofluorescence labelling  Microscopic examination | Freshly isolated thyrocytes expressed APN over the entire plasma membrane.  APN existed on the apical pole of monolayer cells oriented toward the medium.  TSH induced normal follicles formation in isolated thyrocytes suspension.  APN existed on the apical pole of follicular cells facing the follicular lumen. |
| Bell E^20^ | 1984 | Rat | FRTL cells | 3D model  *in vitro* model  *in vivo* model  Thyroid reconstruction | Fibroblasts isolation  FRTL cells and fibroblasts culture  Construction of *TG*E  Thyroidectomy in recipient rats  *TG*E transplantation into rats  Microscopic examination | TGE was constructed *in vitro* when mixing FRTL cells and dermal or thyroid fibroblast simultaneously in matrix medium with additional collagen and hormone supplement.  TGE implanted into thyroidectomized host underwent morphogenesis and formed functional thyroid follicles with vascularization.  TGE did not neither form follicles nor become functional if the host was not thyroidectomized. |
| Nitsch L^21^ | 1984 | Rat | T78 cells | 3D model  *in vitro* model | Monolayer culture of T78 cells  Suspension culture of T78 cells  Morphologic observation of T78 cells  Function assessment of T78 cells  Microscopic examination | T78 cells preserved the morphological and functional characteristics of thyrocytes.  T78 cells formed solid roundish aggregates with small extracellular lumen in suspension culture.  Additional TSH increased the size of follicles in suspension culture.  T78 cells, after 8 months in suspension culture, failed to form follicles. |
| Sho K^22^ | 1984 | Swine | Thyroid tissue | 3D model  *in vitro* model | Thyrocytes isolation  Thyrocytes culture  NaI^131^ incorporation into medium  Microscopic examination  Iodinating activities analysis | TSH induced follicle formation from isolated thyrocytes.  Additional insulin to TSH-containing culture medium stimulated the construction of follicles and the recovery of iodine metabolism of thyrocytes.  Additional insulin alone caused no follicle formation.  The insulin stimulation occurred after a latency period of 24 hours.  Insulin had no growth promoting effect, but might act in concert with TSH in regenerating the characteristic properties of differentiated cells. |
| Kitajima K^23^ | 1985 | Swine | Thyroid tissue | 3D model  *in vitro* model | Thyrocytes isolation  Thyrocytes suspension culture  Electron microscopic examination | Isolated thyrocytes formed inverted follicles with opposite polarities in suspending culture (about 4 days).  Elements of the intercellular tight junction migrated firstly towards culture medium during polarity reversal. |
| Mulcahy R T^24^ | 1985 | Rat | FRTL-5 cells | 3D model  *in vitro* model | Monolayer culture of FRTL-5 cells  Suspension culture of FRTL-5 cells  Microscopic examination | TSH was required for initial cell aggregation and growth from FRTL-5 cells.  TSH induced follicle formation from FRTL-5 cells.  Follicular structures and high cellular activity could be maintained in continuous TSH-supplemented medium. |
| Massart C^25^ | 1988 | Human | Thyroid tissue | 3D model  *in vitro* model | Thyrocytes isolation  Thyrocytes culture inside the collagen  Additional TSH stimulation  Thyroid hormones and *TG* assay  Microscopic examination | Isolated thyrocytes formed follicles in the collagen gel at 4 days.  Newly formed follicles preserved the ability of producing TG and T3, and responded well to TSH.  The detected T3 also derived from T4 deiodination inhibited by PTU. |
| Derwahl M^26^ | 1990 | Rat  Human | FRTL-5 cells  Thyroid tissue | 3D model  *in vitro* model  *in vivo* model  Thyroid reconstruction | Human thyrocytes isolation  FRTL-5 cells culture as monolayer  FRTL-5 cells culture in collagen gel  Human thyrocytes culture as monolayer  H^3^Thymidine injection into nude mice  FRTL-5 cells transplantation into mice  Microscopic examination | Thyrocytes derived from both rat and human formed widely different size of colonies during culture.  The size of thyrocytes cluster was not correlated with the proliferation abilities of cells both *in vitro* or *in vivo*.  Thyrocytes had strong tendency to aggregate in cohorts instead of being scattered randomly throughout each cluster both *in vitro* or *in vivo*.  Thyrocytes *in vitro and in vivo* did not grow individually and independently from each other, but interacted with each other with synchronization. |
| Toda S^27^ | 1990 | Swine | Thyroid tissue | 3D model  *in vitro* model | Thyrocytes isolation  Thyrocytes culture in the collagen  Thyroid hormones and TG assay  Cell proliferation assessment  Microscopic examination | Isolated thyrocytes proliferated in the collagen gel culture.  Isolated thyrocytes reconstructed follicles with morphological polarity peculiar to normal thyroid follicles in the collagen gel culture.  Reconstructed follicular cells produced T3, T4, and TG.  A single cell with a developed cavity underwent cell division, and two cells constructed the primary follicle structure. |
| Sasaki M^28^ | 1991 | Human | Thyroid tissue | 3D model  *in vitro* model | Thyrocytes isolation  Thyrocytes culture in the collagen  Thyrocytes culture between the collagen  Microscopic examination | Isolated thyrocytes formed monolayers on the collagen gel surface.  Isolated thyrocytes formed follicles when cultured in collagen gel or between two layers of collagen gel.  Thyrocytes formed more follicle-like structures with distinct cellular polarity when cultured between two layers of collagen gel.  Thyrocytes formed small lumen follicles with less distinct cellular polarity when cultured in collagen gel. |
| Westermark K^29^ | 1991 | Swine | Thyroid tissue | 3D model  *in vitro* model | Follicles isolation  Follicles culture in collagen gel  Additional EGF and TSH stimulation  H^3^Thymidine labeled incubation  Microscopic examination | Follicles were homogeneously distributed in the collagen gel, either singly or in small aggregates of two to five follicles.  EGF induced a progressive migration of thyrocytes radiating from the preexisting (mother) follicles into the collagen lattice.  EGF-inducible migrating cells were often interconnected with junctional complexes, thus forming small follicles.  Exposure to EGF caused a progressive increase in the number of H^3^ thymidine-labeled nuclei in follicles, indicating active cell multiplication.  EGF might played a role in the process of multiplication and migration of thyrocytes and formation of new follicles in the thyroid. |
| Martin A^30^ | 1993 | Human | Thyroid tissue | 3D model  *in vitro* model  *in vivo* model  Organoid | Thyrocytes isolation  Thyrocytes culture  Thyroid organoid construction  Organoid transplantation into SCID mice  Microscopic examination  Thyroid hormones and TG assay | Thyroid organoid was reconstituted with isolated thyrocytes and Matrigel.  Thyroid organoid showed the formation of multiple reconstituted thyroid follicles with a vascularized solid firm nodule appearance.  Human TG was detected in the serum of thyroid organoid engrafted SCID mice.  TSH increased the human TG levels in the serum of thyroid organoid engrafted SCID mice.  TSH increased thyroid epithelial cell size, enlarged nuclei, and an accompanying decrease in colloid content of follicles in thyroid organoid.  There was no change in thyroid hormone levels between thyroid organoid transplantation SCID mice and normal SCID mice. |
| Valentine M^31^ | 1994 | Human | Thyroid tissue | 3D model  *in vitro* model  *in vivo* model  Organoid | Thyrocytes isolation  Thyrocytes culture  Thyroid organoid construction  Organoid transplantation into SCID mice  Additional T3 supplement to mice  Microscopic examination  Thyroid hormones and TG assay | Thyroid organoid was reconstituted with isolated thyrocytes and Matrigel.  Thyroid organoid showed the formation of multiple reconstituted thyroid follicles with a vascularized solid firm nodule appearance.  Human TG was detected in the serum of thyroid organoid engrafted SCID mice.  There were no apparent differences in morphology and follicle numbers between euthyroid engrafted and hyperthyroid engrafted mice.  The reduced growth factors state in Matrigel, such as EGF and IGF-I, caused no interference with thyroid follicle reconstitution *in vivo*. |
| Bürgi-U^32^ | 1998 | Rat | Thyroid tissue  FRTL-5 cells | 3D model  *in vitro* model | Thyrocytes and follicles isolation  Thyrocytes and FRTL-5 cells culture  Cell culture in alginate bead system  I^125^-labeled substrates incorporation  Cellular proliferation assessment  Microscopic examination | Both isolated thyrocytes and FRTL-5 cells were able to replicate and produce TG in alginate bead culture system.  Isolated thyrocytes could formed follicles, but FRTL-5 cells only formed clusters in alginate bead culture system.  Collagen IV, laminin, perlecan, and fibronectin were identified with positive staining in cultured thyrocytes and FRTL-5 cells. |
| Glaser C^33^ | 1999 | Rat | Newborn  thyroid tissue | 3D model  *in vitro* model  Organoid | Thyroid tissue slice culture  Tissue incorporation in alginate bead system  Different supplements adding to the medium  Cellular proliferation assessment  Microscopic examination | Thyroid organoids were reconstituted form thyroid tissue slice and alginate bead.  Thyroid organoids maintained morphological integrity, functional activity, and ability to proliferate *in vitro*.  Additional iodide or iodide plus TSH to the cultures significantly increased follicular diameters.  Additional of methimazole to the cultures significantly decreased follicular diameters.  Collagen IV, laminin, perlecan, and fibronectin were identified with positive staining in thyroid organoids. |
| Martin A^34^ | 2000 | Human | Thyroid tissue | 3D model  *in vitro* model  Thyroid reconstruction | Thyrocytes isolation  Thyrocytes culture in RCCS  BME and KGF supplement to the medium  *TG* assay  Cell growth assessment  Microscopic examination | RCCS was a suspension culture system generating low-shear environment presented in simulated microgravity.  Levels of TG increased during 2 weeks of simulated microgravity culture.  DNA synthesis of cells increased 150% or more in KGF-treated cultures when exposed to simulated microgravity.  Thyroid organoids were reconstructed using thyrocytes in RCSS only when supplemented with BME and KGF.  Thyroid organoids showed densely arranged multicellular follicles enclosing a central lumen.  Adhesion molecules, such as integrins α3, integrins β1 and E-cadherin, were detected in monolayer thyrocytes, but not in thyroid organoids. |
| Tonoli H^35^ | 2000 | Rat | FRTL cells | 3D model  *in vitro* model | FRTL cells culture  Cx32 and Cx43 cDNA construction  Gene transfection by plasmids  Microinjection of fluorescent probes  Gene and gene expression analysis  Cell proliferation assessment  Microscopic examination | FRTL-Cx43 cell clones exhibited a high level of gap junctions-mediated cell to cell communication compared with wild-type FRTL cells.  Domes were formed from the accumulation of fluid underneath limited areas of the cell layer during monolayer culture.  Cells expressing Cx32 exhibited a much higher capacity to form domes than either wild-type or Cx43-transfected cells.  Domes derived from FRTL-Cx43 cells would transform to closed 3D follicular-like structures with lumen.  These 3D follicular-like structures exhibited an opposite polarity (inside-out polarity).  Cx32, not Cx43 expression by thyrocytes was closely related to the organization of cells into thyroid follicles. |
| Green L M^36^ | 2002 | Rat | FRTL-5 cells | 3D model  *in vitro* model  Thyroid reconstruction | FRTL-5 cells culture in bioreactor vessel  FRTL-5 cells culture in flask  FRTL-5 cells irradiation  Microscopic examination  T4 an TGF-β assays | Bioreactor vessels (Synthecon) was a suspension culture system generating low-shear environment presented in simulated microgravity.  Bioreactor-generated tissue was forming mini-thyroid glands, as evidenced by multiple follicles presented in a single mass.  The cytoskeletal components, F-actin and microtubules, in bioreactor-engineered tissue were disrupted following irradiation.  The cytoskeletal components in flask grown cultures appeared slightly affected following irradiation, and returned to normal after 24 hours.  The level of T4 in the supernatant was reduced following irradiation.  The level of TGF-β in the supernatant was increased following irradiation. |
| Toda S^37^ | 2002 | Swine | Thyroid tissue | 3D model  *in vitro* model | Minced thyroid tissue of 1mm in diameter  Air-exposure (AE) culture system establishment  Morphology and morphometric analysis  Immunohistochemistry examination  ATP assay  Western blotting for mATP synthase-α | A new organotypic AE culture method of thyroid tissue maintaining 3D follicles with C cells for more than one month was depicted.  AE-treated culture preserved thyroid follicles from death, while most of the tissues became necrotic and died by 7 days in AE-untreated culture.  Most of the thyroid cells displayed *TSHR*, *NKX2-1*, *TG* and pendrin in the AE-treated culture.  The total amount of cellular ATP in culture with or without AE gradually decreased in a time-dependent manner.  At 72 hours, cellular mATP synthase-α was expressed more strongly in an AE condition than in a non-AE state.  TSH did not affect the survival of the thyroid tissues, cellular ATP amount or mATP synthase-α expression with or without AE. |
| Toda S^38^ | 2003 | Swine | Thyroid tissue | 3D model  *in vitro* model | Minced thyroid tissue of 1mm in diameter  AE culture system establishment  Tissue culture in AE system  Microscopic examination  Cell growth assessment  Immunohistochemistry examination  C cell number and growth assessment | New folliculogenesis formed in the peripheral zones of tissue, and preexisting follicular structures changed little in central zones of tissue during culture.  Three types of follicle formation from (preexisting)mother follicles were named the solid nest type, the budding type, and the lumen-dividing type.  The frequencies of solid nest, budding and lumen-dividing types of follicle formation were approximately 20%, 40% and 40%, respectively.  Additionally, a few single or clustered thyrocytes in peripheral zones, reconstructed small follicles through isolated cell-derived folliculogenesis.  The BrdU uptake of thyrocytes was prominently higher in the peripheral zones of the embedded tissues than in the center.  C cells were rarely observed in the newly formed follicles, and showed neither hyperplasia nor BrdU incorporation during culture. |
| Lin R Y^39^ | 2003 | Mouse | ESCs | 3D model  *in vitro* model | ESCs culture  EBs formation, culture, and differentiation  Additional TSH stimulation  RNA isolation and gene expression analysis  Immunofluorescent microscopy  Intracellular cAMP measurement | *TSHR* positive cells were firstly seen in 6 days EBs, then were *PAX8* and *TTF2* positive cells in 8 days EBs.  ESCs-inducible EBs could express *TPO*, *PAX8*, *TG*, *TPO*, and *TSHR*, indicating ESCs had the potential to differentiate into the thyrocyte lineage *in vitro*.  EBs-derived *TSHR* positive cells showed a higher intracellular cAMP content when treated with TSH, indicating *TSHR* presented in these cells can mediate a signal.  *PAX8* and *TSHR* mRNA transcripts were robustly expressed in EBs-derived cells grown in the presence of TSH.  TSH was necessary to maintain the expression of *PAX8* and *TSHR* genes during EB differentiation. |
| Arufe M C^40^ | 2006 | Mouse | ESCs | 3D model  *in vitro* model | *TSHR* (^+^/^–^) ESCs development  ESCs culture  ESCs differentiation  EBs formation, culture, and differentiation  Cell sorting and gene expression analysis  Immunofluorescent staining  Radioactive iodide uptake | A fusion gene with GFP-Neo^r^ under the control of *TSHR* promoter was electroporated into ESCs.  Inactivation of one allele of the *TSHR* gene did not alter the developmental program during EB differentiation.  *TSHR* heterozygosity (*TSHR*^+^/^–^) did not affect the *in vitro* differentiation potential of ES cells.  GFP expression faithfully mimicked endogenous *TSHR* expression in differentiating EBs, and can be easily measured and quantified.  TSH can stimulate *TSHR* expression in EBs in the absence of serum.  After 21 days of differentiation, the cells treated with TSH showed iodide uptake activity.  TSH induced the thyroid follicular cells developmental program derived from EBs *in vitro*. |
| Hoshi N^41^ | 2007 | Mouse | Thyroid tissue | 3D model  *in vitro* model | Follicles and thyrocytes isolation  Hoechst 33342 and antibody staining for FACS  Cell sorting and culture in collagen gel.  Histochemical analyses  Gene expression analysis by qRT-PCR | SP cells were present in mouse thyroid, ranging from 0.3–1.4% the total population of cells.  FACS analysis revealed that CD45+ cells constituted only approximately 3% the SP population of cells, whereas SCA1+ cells represented approximately 56%.  Thyroid SP cells were subfractioned to two population of cells: *SCA1+/CD45–/CD117–*and *SCA1–/CD45–/CD117–*.  Base on gene expression analysis, SP cells were less differentiated or not terminally differentiated and had characteristics of a stem/progenitor cell.  *ABCG2*+ cells were in good agreement with the number of Hoechst-effluxing SP cells, and they were detected only in the interfollicular space, but not in follicles.  When cultured in collagen gel system in vitro, only MP cells, but not SP cells, developed functional thyroid follicles. |
| Lan L^42^ | 2007 | Human | Thyroid tissue | 3D model  *in vitro* model | Thyrocytes isolation and culture  SP cells isolation with FACS  Growth and differentiation of thyrosphere  BrdU incorporation in thyrosphere cells  Gene expression analysis by sqRT-PCR  Iodide uptake assay for sphere-derived cells | Thyroid adult stem cells were isolated by FACS as SP cells from human thyroid.  SP cells strongly expressed *ABCG2* and *OCT4* which were major transcription factors for embryonic and some adult stem cells.  SP cells were negative for thyroid differentiation markers, such as *TG*, *TPO*, *PAX8*, *TSHR* and *TPO*.  Viable SP cells were maintained in culture for up to 14 days. However, neither cell attachment nor growth was observed in monolayer culture or in Matrigel.  A significant proliferation rate was also absent when side population cells were cocultured with normal thyrocytes in a two-chamber culture system, even with EGF.  Isolated side population cells cultured as monolayer or in Matrigel were resistant to growth stimulation due to lack of interaction between stem cells and niche cells.  Proliferative stem cells could be isolated directly from primary thyroid cultures as nonadherent, three-dimensional spheres in a medium enriched with EGF and BFGF, with a complementary approach that was first used to separate neural stem cells.  In response to TSH and serum, sphere-derived stem cells differentiated into thyroid cells expressing *PAX8*, *TG*, *TPO*, *TSHR*, and *TPO*, and showed TSH-dependent I125 uptake. |
| Fierabracci A^43^ | 2008 | Human | Thyroid tissue | 3D model  *in vitro* model  *in vivo* model  thyroid organoid | Thyroid tissue digestion  Tissue culture in specific “spheroid medium”  Sphere formation, dissociation and culture  Proliferation assay of thyrosphere  Telomerase gene expression analysis  Telomere length analysis  Clonogenesis  Immunophenotyping  Expression analysis of nestin, OCT4 and Nanog  Morphological and functional assessments  *in vivo* tumorigenicity assessment | Small aggregates of cells floating in the “spheroid medium” proliferated rapidly and appeared with a spheroid-like structure, named thyrosphere.  The morphology of cells forming thyrosphere were different from adult mature thyrocytes with large nucleus and narrow cytoplasmic ring.  Thyrosphere cells showed most high proliferation abilities when adding the mixture of growth factors (EGF and BFGF) together to the culture medium.  The expression of telomerase was undetectable and the telomere length shortened during thyrospheres culture.  Thyrospheres generated from the thyroid specimens were found to be composed of clonally derived cells and did not simply represent cellular aggregates.  By immunocytochemistry, a population of cells within spheres were positive for *TG*, but no cells were positive for *TPO* or *TSHR* in seven generated lines.  By FACS, intracytoplasmic TG and TPO were positive in 4 of 9 generated lines, but no staining for TSH-R and TPO was detected in all generated lines.  Surface stem cell marker CD34 was identified in 1–25% subpopulation of cells, CD45 and CD117 were both negative.  The expression of *Nestin*, *OCT4* and *Nanog* was detected positive in 8, 5, 5 of 12 generated lines, respectively.  Thyrosphere seeded in collagen gel in the presence of “differentiation medium” started to generate functional thyroid follicles.  Thyrosphere seeded in collagen gel in the presence of “spheroid medium” did not differentiate into follicles, but maintained a spheroid-type morphology.  T4 progressively increased in the supernatant of the spheroids cultured in “differentiation medium”, while T3 and T4 were not detected in spheroid culture medium.  Thyrosphere injected into SCID mice did not develop tumors within the period of observation of 2 months, either subcutaneously or intra-organ.  Thyrosphere showed an extent of plasticity when co-cultured with the neuroblastoma cell line and the adipogenic lineage. |
| Arauchi A^44^ | 2009 | Rat | Thyroid tissue | 3D model  *in vitro* model  *in vivo* model  Thyroid reconstruction | Cell culture surface preparation with PIPAAm  Thyroid cells isolation and culture  Thyroid cell sheets preparation  Total thyroidectomy-inducible hypothyroid rats  Thyroid cell sheets transplantation into rats  Microscopic examination  Thyroid hormones assay | The diameter and thickness of thyroid cell sheets cultured for 1 week from temperature-responsive culture dishes were about 10mm and 50um, containing 1×10^7^ cells.  In the center of thyroid cell sheets cultured in vitro, typical thyroid follicles storing colloid were found with positive *TTF1* immunostaining in follicular inner surface.  Transplanted thyroid cell sheets became obviously thicker *in vivo* than in vitro, and organized into honeycomb-like structures of thyroid follicles.  Thyroid cells sheets *in vivo* were similar to native thyroid, consisted of follicles with colloid, parafollicular cells and microvessels containing red blood cells.  FT3 and FT4 increased in all cell sheets transplantation rats in a time-dependent manner.  In the 1/4 size cell sheet transplantation group, although FT3 and FT4 levels became saturated at 4 weeks after transplantation, they did not reach the normal levels. |
| Antonica F^45^ | 2012 | Mouse | ESCs | 3D model  *in vitro* model  *in vivo* model  Thyroid organoid | *NKX2-1*+ and *PAX8*+ ESCs generation  EBs formation, culture, and differentiation  Gene expression analysis by qRT-PCR  Immunodetection  I^131^-inducible hypothyroid mice model Transplantation of thyroid organoid  Iodide organification assay  Thyroid hormones assay | Exogenous *NKX2-1* and *PAX8* expressions in mouse ESCs were temporarily induced with a Tet-On system by adding doxycycline into the medium for 3 days.  Forced transient co-expression of *NKX2-1* and *PAX8* induced the auto-induction of endogenous *NKX2-1* and *PAX8* expression in ESCs.  Doxycycline-treated ESCs cannot directly differentiate into follicular cells, suggesting additional factors might be required to promote follicular morphogenesis.  When treated with additional TSH, these doxycycline-treated *NKX2-1*+ and *PAX8*+ ESCs differentiated into thyroid follicular cells.  *NKX2-1*+ and *PAX8*+ ESCs-derived thyroid follicular cells organized into 3D follicular structures, and showed appreciable iodide organification activity.  At the grafting site, numerous follicles were surrounded by a dense network of micro-vessels, demonstrating the formation of classical angio-follicular units.  The differentiation protocol did not promote C cells development.  Four weeks after grafting, hypothyroid mice presented a substantial increase in plasma T4 level, and a full normalization of body temperature, indicating these grafting thyroid organoids had potent functional capacity to compensate for the lack of orthotopic thyroid tissue. |
| Ozaki T^46^ | 2012 | Mouse | Thyroid tissue | *in vivo* model  Thyroid regeneration | Partial thyroidectomy  Histological examination  Electron microscopic examination  Cell sorting by laser capture microdissection  RNA preparation and microarray analysis  Serum T4 and TSH assays | The central part of the intact thyroid lobe where many microfollicles were present generally served as a center for proliferation.  The number of microfollicles and/or cells with clear or faintly eosinophilic cytoplasm (clear cells) markedly increased in the proliferative area after thyroidectomy.  These clear cells were immature cells which would participate in the repair and/or regeneration of the thyroid gland.  The proliferative center extended to the area near the cut edge after partial thyroidectomy.  Thyroidectomy affected a whole thyroid lobe, resulting in markedly disorganized physiology of cells with altered cellular and/or metabolic status, and some of the changes may resemble to those seen during thyroid gland development.  Among the top genes affected in the proliferative center after thyroidectomy, many were involved in cancers.  TSH level declined and could not return to baseline level, whereas T4 level was basically normal at two weeks after thyroidectomy.  *Krt14* was barely detected in normal thyroid, whereas after surgery, intense staining for *Krt14* was identified in a restricted area within the proliferative area. |
| Ma R^47^ | 2013 | Mouse | ESCs | 3D model  *in vitro* model | ESCs culture  *NKX2-1* and/or *PAX8* gene transfection in ESCs  EBs formation, culture, and differentiation  Gene expression analysis by sqRT-PCR  Immunodetection | In undifferentiated ESCs, overexpression of *NKX2-1* and *PAX8* did not change the pluripotent state of ESCs, since major stemness markers continued to be expressed.  In undifferentiated ESCs, co-expression of *NKX2-1* and *PAX8* induced robust expression of *TPO*, *TSHR*, *TG*, and *TPO*. However, the degree of thyroid-specific genes induction remained limited when compared to the control thyroid cells (FRTL-5).  In undifferentiated ESCs, co-expression of *NKX2-1* and *PAX8* initiated a change in the cell’s fate toward a thyroid follicular cell lineage, but appeared to be insufficient for full thyrocyte fate determination.  With the removal of LIF and the supplements of activin A plus TSH, *NKX2-1*+/*PAX8*+ ESCs differentiated into mature thyroid follicular cells with high expression of thyroid-specific genes (*TPO*, *TSHR*, *TG*, and *TPO*).  On continued *in vitro* culture, these cells formed 3D thyroid follicles and presented TG protein in the follicular lumen. |
| Okamato H^48^ | 2013 | Mouse | Thyroid tissue | *in vitro* model  *in vivo* model  Thyroid regeneration | R26R; *TPO-Cre* mice generation  Partial thyroidectomy  BrdU incorporation  Histological observations  Immunohistochemistry observations  Immunofluorescence observations  Thyroid tissue culture in collagen gel | R26R; *TPO-Cre* mice were generated through crossing *ROSA26-β-Gal* transgenic mice with *TPO-Cre* transgenic mice.  After partial thyroidectomy, *SCA1* and *BrdU* positive cells in the thyroid were negative for *β-gal*, *NKX2-1*, *CD34*, *CD133*, *Oct4* and *SOX10*, suggesting these cells were not of thyroid follicular cell, hematopoietic cell, ESCs, adult stem cell and neural crests origin.  High *SCA1* expression was detected in irregular follicles with no *β-gal* expression in the area close to the cut edge at 35 days after surgery.  *SCA1/BrdU* positive and *β-gal/**NKX2-1* negative cells would become *SCA1/BrdU/β-gal/NKX2-1* positive cells at 120 days after surgery.  *SCA1* positive cells might be responsible for repair and/or regeneration of thyroid follicular cells after damage caused by partial thyroidectomy.  *Krt14*+ cells were demonstrated with no specificity as they could found in both follicular cells and non-follicular areas. |
| Kurmann A A^49^ | 2015 | Mouse  Human | ESCs/iPSCs  Fibroblasts | 3D model  *in vitro* model  *in vivo* model  Thyroid organoid | *NKX2-1^GFP^* ESC/iPSC mouse lines generation  *NKX2-1^mCherry^* ESC/iPSC mouse lines generation  EBs formation, culture, and differentiation  *NKX2-1^GFP^/PAX8^tdTomato^* iPSC lines generation  Human dermal fibroblasts-derived iPSCs lines  Directed differentiation of human iPSCs  I^131^-inducible hypothyroid mice model  Cell preparation for *in vivo* transplantation  Kidney capsule transplantation surgery  Blood chemistry and hormonal analyses | Microarray analysis revealed multiple active signaling pathways, including *BMP*, *WNT*, *EGF*, and *FGF* signaling, in ESC-derived *NKX2-1^GFP^* endodermal progenitors  The essential factors for thyroid cell development were identified by sequential withdrawal of each individual factor from the *WFKBE*+*2* cocktail.  BMP4 and FGF2 were sufficient to specify endodermal cells *in vitro* toward *NKX2-1*+ endodermal progenitor cells with thyroid potential.  *NKX2-1* haploinsufficiency did not affect specification of *NKX2-1*+ endodermal cells, but did affect subsequent maturation of thyroid epithelial cells.  *NKX2-1^GFP+^* cells were competent to subsequently express thyroid differentiation markers after BMP4 and FGF2 induction period.  The thyroid lineage was specified in a distinct *NKX2-1*+ endodermal population in the developing embryo.  BMP4 and FGF2 was necessary and sufficient for thyroid lineage specification from mouse and xenopus anterior foregut endoderm or ESCs-derived endoderm.  Addition of TSH after lineage specification resulted in increased *TPO*, *TSHR*, and *TPO* expression.  Culture of purified *NKX2-1mCherry*^+^ cells in 3D Matrigel conditions triggered the formation of follicular-like clusters of cells.  ESC-derived thyroid follicular cells prepared by directed differentiation exhibited *in vivo* functional potential, including the capacity to rescue hypothyroid mice.  The capacity of combinatorial BMP and FGF signaling to induce thyroid lineage specification from developing endoderm across species, from Xenopus to mice to humans, and enabled the generation of patient-specific thyroid progenitors from individuals with genetic or congenital hypothyroidism. |
| Ma R^50^ | 2015 | Human | ESCs (line H9) | 3D model  *in vitro* model | ESCs culture  *NKX2-1* and/or *PAX8* gene transfection in ESCs  EBs formation, culture, and differentiation  Gene expression analysis by RT-PCR  Immunodetection  *TSHR* functional assessment  Radioactive iodide uptake | Stable *PAX8*+ and/or *NKX2-1*+ human ESCs lines were generated through virus transduction by coculture ESCs and lentivirus carrying target genes.  *PAX8* and/or *NKX2-1* gene transfection did not change the pluripotent state of ESCs, since major stemness markers continued to be expressed.  In undifferentiated *NKX2-1*+ and/or *PAX8*+ human ESCs lines, *TPO* and *TSHR* protein were detected with the exclusion of *TG*.  In ultra-low attachment dishes culture, when treated with activin A and TSH, double transfected ESCs-derived EBs differentiated into thyrocytes with thyroid-specific gene expressions.  Differentiated *NKX2-1*+ and *PAX8*+ ESCs were capable of forming 3D thyroid follicles with *TG* protein in the follicular lumen, and expressed thyroid-specific genes (*TPO*, *TSHR*, *TG*, and *TPO*).  These differentiated thyroid cells could generate cAMP, uptake I^125^, respond to TSH and inhibit by sodium perchlorate. |
| Ma R^51^ | 2015 | Mouse | iPSCs | 3D model  *in vitro* model  *in vivo* model  Thyroid organoid | iPSCs generation by MEFs reprograming  *NKX2-1* and/or *PAX8* gene transfection in iPSCs  EBs formation, culture, and differentiation  Gene expression analysis by qRT-PCR  Immunofluorescence staining  *TSHR* functional assessment  Radioactive iodide uptake  Thyroid follicle formation *in vivo* | iPSCs were generated by reprograming EFs transfected with a “stem cell cassette” lentiviral vector composing *OCT4*, *KLF4*, *SOX2* and *CMYC.*  iPSCs displayed the typical morphology of ESCs and expressed classical ESCs markers such as *OCT4*, *SOX2*, *Nanog*, and *REX1*.  *PAX8*+ and/or *NKX2-1*+ iPSCs were generated by electroporating the *PAX8*+ and/or *NKX2-1*+ lentiviral vectors into iPSCs.  Overexpression of *NKX2-1* and *PAX8* did not change the pluripotent state of iPSCs, since major stemness markers continued to be expressed.  In undifferentiated iPSCs, co-expression of *NKX2-1* and *PAX8* initiated a change in the cell’s fate toward a thyroid follicular cell lineage, but appeared to be insufficient for full thyrocyte fate determination.  In ultra-low attachment dishes culture, when treated with activin A and TSH, double transfected iPSCs-derived EBs differentiated into thyrocytes with thyroid-specific gene expressions.  These differentiated thyroid cells could generate cAMP, uptake I^125^, respond to TSH and inhibit by sodium perchlorate.  Formation of thyroid tissue in the host mice with TG deposition in the follicular lumen were demonstrated after subcutaneously injection of *NKX2-1*+/*PAX8*+ iPSCs following Activin A exposure for 5 days into nude mice. |
| Antonica F^52^ | 2017 | Mouse | ESCs | 3D model  *in vitro* model  *in vivo* model  Thyroid organoid | *NKX2-1*+ and *PAX8*+ ESCs culture  EBs formation  EBs embedding in 3D Matrigel  Induced thyroid differentiation  Transplantation of thyroid organoid  Analysis of the grafting | A valid protocol for the generation of functional thyroid tissue using mouse ESCs was provided.  All the details and references for ESCs-derived thyroid organoid characterization and analysis both *in vitro* and *in vivo* were given. |
| Bulanova E A^53^ | 2017 | Mouse | Embryonic thyroid tissue  Embryonic allantoic tissues | 3D model  *in vitro* model  *in vivo* model  Thyroid reconstruction | Thyroid tissue microdissection from E14.5 MB  Allantoic tissue microdissection from E8.5 MB  Generation of TS and AS in hanging drop culture  Kinetics analysis of spheroids fusion  Bioprinting thyroid constructs  Culture of printed thyroid constructs  Immunofluorescence examination  I131-inducible hypothyroid mice model  Transplantation of bioprinted thyroid constructs  Thyroid function assessment *in vivo* | Mouse thyroid gland was reconstructed from embryonic thyroid tissue-derived TS and embryonic allantoic tissue-derived AS in collagen gel culture using a multifunctional Fabion 3D bioprinter with the turnstile system.  TS and AS had the property of endogenous fusion, thereby validating their use as building blocks for thyroid bioprinting.  In printed constructs containing only TS, ECs were only found at the periphery of thyroid tissue after 4 days in collagen culture.  In printed constructs containing both TS and AS, ECs displayed abundantly and localized around the epithelial cells of thyroid tissue after 4 days in collagen gel.  Endothelial progenitors in AS responded and were attracted by the angiogenic factor, VEGF-A, highly secreted by thyrocyte progenitors of the TS.  Addition and fusion of AS to TS within collagen hydrogels allowed improved vascularization of bioprinted TS.  3 and 5 weeks after grafting three TS and six AS, recipient hypothyroid mice showed a gradual normalization of body temperature and a substantial elevation of serum T4 level, indicating bioprinted thyroid construct was functional.  At the grafting site, numerous thyroid follicles with new capillary formation filled with erythrocytes were present at the renal cortical area of mice. |
| Lee J^54^ | 2017 | Mouse | Thyroid tissue | *in vivo* model  Thyroid regeneration | Bilateral lower third partial thyroidectomy  Histological examination  Thyroid hormones assay  Thyroid gland activation analysis  Immunohistochemical analysis | The remaining thyroid gland could not produce a sufficient amount of T4 at 7 days after bilateral lower third thyroidectomy.  FT4 returned to normal values, but TSH was significantly elevated at 28 days after bilateral lower third thyroidectomy.  Primitive cells were defined as multiciliated follicular cells with coarsely vacuolated cytoplasm and large vesicular nuclei.  These characterized primitive cells located in the center of the intact lateral lobe of the thyroid gland at 10 days after surgery.  Primitive follicles also existed at the cutting margin of the partial thyroidectomy site at 28 days after surgery.  Follicle formation from primitive follicles was the budding type of mother follicle-derived folliculogenesis.  Primitive thyroid follicles (mother follicles) expressed *LGR4/GPR48* and *FOXA2*, but not expressed *NKX2-1* and *PAX8*.  Primitive thyroid follicles underwent a regeneration process to produce new follicles. |
| Yang Y^55^ | 2017 | Swine | Thyroid tissue | 3D model  in vitro model  Thyroid reconstruction | Follicles and thyrocytes isolation  Follicles and thyrocytes culture  Thyroid cells characterization  Microencapsulation of thyroid cells  Morphological observation  Viability and proliferation analysis  Immunofluorescence examination  Thyroid hormones assay | Microencapsulation of thyroid cells was successfully performed using a newly designed 8-nozzle microfluidic device.  The permeability of alginate-PLO-alginate microcapsule was suitable for smaller molecules to pass through capsule membrane, but not larger molecules, such as immunoglobulin.  The APA microcapsules exhibited a regular round shape with relatively uniform size, and the spheres of porcine thyroid cells were observed in each microcapsule.  With TSH stimulation by TSH, thyroid cells in each microcapsule gathered and formed 3D follicular spheres in the inner core of the microcapsules within 48h.  The viability of the cells in the microcapsules was maintained for at least 28 days in culture, and the proliferation rate of cells was low.  Thyroxine secretion was significantly higher in encapsulated cells than in monolayer cultured cells in complete growth medium. |
| Saito Y^56^ | 2018 | Mouse | Thyroid tissue | 3D model  *in vitro* model  *in vivo* model  thyroid organoid | Thyroid follicles and thyrocytes isolation  Thyroid organoid formation and culture  Functional assessment of thyroid organoid  I131-inducible hypothyroid mice model  Transplantation of thyroid organoid  Tumor formation in p53KO thyroid organoid | Isolated cells formed thyroid organoids when embedding them into organoid culture medium containing Matrigel and various growth factors.  Each thyroid organoid was derived from a single thyroid cell when coculturing allogeneic thyroid cells.  TSH significantly increased both the number of organoids and the number of cells in a concentration-dependent manner.  The established thyroid organoids possessed a follicular structure with TG deposition in the luminal compartment.  The established thyroid organoids maintained TSH-regulated thyroid functions including iodide uptake, and the production and release of thyroid hormone in vitro.  The transplanted thyroid organoids manifested deposition of TG in the follicular colloid and maintained the function of iodide uptake *in vivo.* |
| Alfieri M^57^ | 2019 | Rat | Thyroid tissue | 3D model  in vitro model | Decellularization of rat thyroid  Analysis of the residual DNA content  Trypsin digestion of 3D stromal matrices  Mass spectrometric analysis  Evaluation of collagen type | Three different protocols with detergents and enzymes for rat thyroid gland decellularization were provided.  All three decellularization protocols preserved both the outer fibrous thyroid capsule and the 3D internal structure of the follicles.  A different distribution of collagen types (ECM proteins) was apparent in dependence on the decellularization protocol. |
| Pan J^58^ | 2019 | Rat  Rat  Human  Human | Thyroid tissue  FRTL-5 cells  Thyroid tissue  Parathyroid tissue | 3D model  in vitro mode  Thyroid reconstruction | Perfusion decellularization of rat thyroid  Microscopic examination  Seeding of decellularized thyroid scaffold  Recellularization of seeded thyroid construct  Culture of seeded thyroid construct | Rat thyroid decellularization was achieved by thyroid artery perfusion with 1% sodium dodecyl sulfate.  A rat thyroid-shape translucent acellular scaffold with intact follicular basement membrane and arterial elastic fiber network was obtained after decellularization.  The decellularized thyroid ECM scaffold showed reliable cellular compatibility as ADMSCs could attach and replicate in the matrix.  Seeding FRTL-5 cells into the decellularized scaffolds resulted in cells adherence throughout the scaffold.  *TG* and *TPO* were both expressed in FRTL-5 cells in the recellularization of scaffolds, indicating maintained expression of critical genes.  When co-seeding human thyroid cells and parathyroid cells into the rat decellularized matrix, these cells were capable of distributing throughout the scaffold.  *TG*, *TPO*, and *PTH* expression were all detected positively in this mixed cell organ. |
| Deisenroth C^59^ | 2020 | Human | Thyroid tissue | 3D model  *in vitro* model | Thyrocytes isolation  Thyrocytes culture  Microscopic observation  Immunodetection  Gene expression analysis by qRT-PCR  Thyroid hormone and TG assay | Key expression markers, *NKX2-1*, *Krt7*, and *TG*, were all detected in the human thyrocytes in early passage cells during 2D monolayer culture.  Thyrocytes seeding onto a Matrigel hydrogel showed significant follicle-like self-assembly into small microtissues.  *PAX8*, *NKX2-1*, and *FOXE1* expressions differed from 2D culture and 3D culture, which indicated culture format affected phenotype.  The microtissues presented in the 3D model were more responsive to TSH exposures and expressed genes critical for thyroid hormone production  Neither T3 nor T4 were observed in 2D cultures, indicating deficiency for key hormone production components in this culture format.  3D microtissue culture model for long-term in vitro monitoring of thyroid hormone biosynthesis was feasible and suitable. |
| Jiang D^60^ | 2020 | Human | Thyroid tissue | 2D model  *in vitro* model | Thyrocytes isolation  Thyrocytes culture  Knockdown of *TTF* by siRNA transfection  Incorporation of 4-thiouridine  Gene expression analysis by qRT-PCR  Measurement of mRNA degradation for thyroid-specific genes  Measurement of newly transcribed mRNA  Immunodetection | TSH regulation of thyroid-specific gene expression in human thyrocytes in primary culture was biphasic causing an inverted U-shaped dose-response curve.  Thyroid-specific gene expressions were progressively increased at low TSH concentrations and inhibited at high TSH concentrations.  Knockdown of *NKX2-1* and *FOXE1* inhibited the TSH-induced increase in all genes (*TG*, *TPO*, *DIO2*, *TPO* and *TSHR*).  Knockdown of *PAX8* inhibited the TSH-induced increase in *TG*, *TPO*, DIO2, and *TPO*, but not of *TSHR*.  Knockdown of *HHEX* did not inhibit TSH responses of any of the thyroid-specific genes.  The inverted U-shaped dose-response curve of regulation by TSH of thyroid genes expression was caused by changes in their transcription without any effects on mRNA degradation.  TSH regulation of *NKX2-1*, *FOXE1* and *PAX8* exhibited monophasic dose-response curves with downregulation at high doses of TSH.  Knockdown of *FOXE1*, *NKX2-1*, *PAX8* or *HHEX* did not downregulate the mRNA levels of other *TTF*s. |
| Ran Q^61^ | 2020 | Mouse | ESCs | 3D model  *in vitro* model  *in vivo* model  Thyroid reconstruction | *FGF10 EX1wild/mut* mice generation  *FGF10 EX3wild/mut* mice generation  *FGF10 EX1mut /EX3mut* mice by intercrossing  Blastocyst complementation technique  *FGF10 EX1mut /EX3mut* chimeric mice with GFP+ ESCs  Histological analysis  Contrast-enhanced microcomputed tomography  Thyroid function assay | *FGF10 EX1wild/mut* and *FGF10 EX3wild/mut* mice were generated using CRISPR/Cas9 system.  *FGF10 EX1mut /EX3mut* mice were obtained by intercrossing FGF10 EX1wild/mut with *FGF10 EX3wild/mut* mice.  *FGF10 EX1mut /EX3mut* chimeric mice were obtained by GFP+ ESCs microinjection into the embryo of *FGF10 EX1mut /EX3mut* mice via blastocyst complementation.  The thyroids of neonatal *FGF10 EX1mut /EX3mut* mice were normally shaped but smaller than those of *FGF10wild /wild* neonates.  These hypoplastic thyroids of *FGF10 EX1mut /EX3mut* mice had a lower proportion of parenchyma, decreased branching, and fewer follicles than normal thyroids.  The number of thyroid cells expressing *NKX2-1* and *PAX8* were decreased in neonatal *FGF10 EX1mut /EX3mut* mice.  The total expression levels of *TG* and T3 were reduced in in neonatal *FGF10 EX1mut /EX3mut* mice with the exception of calcitonin.  The thyroids of neonatal *FGF10 EX1mut /EX3mut* chimeric mice were histologically and functional normal, as in neonatal FGF10wild /wild mice.  The thyroids of adult *FGF10 EX1mut /EX3mut* chimeric mice were histologically normal, as in adult *FGF10wild /wild* mice.  *NKX2-1*+, *FOXE1*+, and *PAX8*+ follicular cells of adult chimeric mice were GFP+, indicating these cells were derived principally from GFP+ ESCs.  The extent of GFP expression in non-follicular regions, including parafollicular cells, did not differ between chimeras.  Expression of *TG*, plasma T3 and T4 of adult *FGF10 EX1mut /EX3mut* chimeras were all similar to normal, indicating the thyroids were functional. |
| Ma R^62^ | 2021 | Mouse | Thyroid tissue | 3D model  *in vitro* model  *in vivo* model  Thyroid regeneration | *TPOCreER2* transgenic mice generation  *TPOCreER2*/*iDTR* transgenic mice generation  Intraperitoneal injection of TM and DT  Histology analyses  Thyroid function testing  Gene expression analysis | *TPOCreER2* transgenic mice expressed *Cre-ERT2* under the direction of the *TPO* promoter requiring tamoxifen induced deletion of floxed stop sequences.  *ROSA26iDTR* mice had *Cre*-inducible expression of *iDTR* rendering them susceptible to ablation following diphtheria toxin administration.  *TPOCreER2*/*iDTR* mice were generated by intercrossing ROSA26*iDTR* mice with *TPOCreER2* mice which could serve as a model for depletion of *TPO*-expressing thyroid gland cells.  Serum T4 markedly decreased and TSH increased after 4 weeks of TM/DT administration in *TPOCreER2*/*iDTR* mice.  Serum T4 restored to basal levels and in parallel TSH levels decreased after additional 8 weeks recovery in these hypothyroid mice.  More than 90% *TPO*-expressing mature thyroid follicles were damaged after 4 weeks of TM/DT administration.  Thyroid gland recovered with the formation of new thyroid follicles as early as 2 weeks and appeared as normal glands 8 weeks post treatment.  Gene expression analysis revealed expressions of stem cell markers significantly increased in thyroid glands during and following TM/DT administration. with a transient surge in all stem cell markers at 2 weeks after TM/DT administration.  Resident thyroid progenitor cells with positive *PAX8* expression and negative *TG* expression were found in thyroid glands. |
| Ogundipe V M L^63^ | 2021 | Mouse  Human | Thyroid tissue  Thyroid tissue | 3D model  *in vitro* model  *in vivo* model  Thyroid organoid | Thyrocytes isolation  Mouse thyrocytes culture in TGM  Human thyrocytes culture in TGM with WR  In vitro self-renewal assay  In vitro cell cycle analysis  Dual pulse labelling for asymmetric division  In vitro differentiation assay  I131-inducible hypothyroid mice model  Transplantation of thyroid organoid  Tumorigenic potential testing  Gene expression analysis by microarray  Immunodetection | Both murine and human isolated thyroid cells formed thyrospheres when cultured in specific thyroid gland medium with WR or not.  Thyroid specific genes (*NKX2-1*, *PAX8*, *TG*, *TSHR*, *TPO*) were all expressed by both murine and human thyroid gland-derived thyrospheres.  Both WNT and R-Spondin1 were additionally required in the TGM to establish and propagate human organoid cultures through single thyrosphere-derived cells.  Comparing human- and murine-derived cells, a higher organoid-forming efficiency was displayed by the murine-derived culture.  Human thyroid organoids expressed *NKX2-1*, *PAX8*, and *TPO*, and maintained the phenotype from their tissue of origin during passaging.  Both murine and human organoids were able to maintain and/or redevelop thyroid epithelium integrity.  Proliferating putative stem cells existed in both murine and human organoids, although a distinctive thyroid stem cell marker was not identified.  Both murine and human organoids had follicular structures and expressed differentiation markers in Matrigel with thyroid maturation medium.  Both murine and human thyroid-derived cells were able to form organoids capable of in vitro self-renewal and differentiation.  When thyroid-derived cells were transplanted into mice, they could self-renew into organoids and grow to mature state with time.  Thyroid tissue-derived organoids produced FT4 *in vivo*, but the process was very modestly, perhaps due to the limited cell numbers.  Tumor-related markers of these thyroid organoids were not induced even after irradiation and long-term culturing. |
| Romitti M^64^ | 2021 | Mouse | ESCs | 3D model  *in vitro* model  Thyroid organoid | *NKX2-1*+/*PAX8*+/*TG*+^EGFP^ ESCs generation  ESCs culture  EBs formation, culture, and differentiation  FACS analysis of thyroid organoid  scRNAseq data analysis of thyroid organoid  Pharmacological inhibition of *TGF-β* pathway  Gene expression analysis by qRT-PCR  Immunodetection  Iodide organification assay | Three gene constructs were engineered into the ESCs, which allowed doxycycline-induced expression of exogenous *NKX2-1* and *PAX8* and monitoring of the thyroid lineage by *TG*-driven EGFP expression.  Exogenous *NKX2-1* and *PAX8* expressions in ESCs were temporarily induced with a Tet-On system by adding doxycycline into the medium.  Thyroid follicular cells were mature by day 22, expressed thyroid transcription factors (*NKX2-1*, *PAX8*, HHEX, and *FOXE1*) as well as functional markers (*TG*, *TSHR*, *TPO* and *TPO*), and self-organized into 3D follicular structures containing iodinated *TG* in the lumen.  Nine cell clusters, including ESCs, endothelial cells, immune cells, mesodermal cells, fibroblast cells, neuronal-like cells, non-thyroid cells, and doxycycline-responsive cells and mature thyrocytes, were identified from ESCs-derived thyroid organoid through scRNAseq analysis.  Gene ontology revealed involvement of distinct metabolic and biological processes to each cell type.  Although thyrocytes uniformly expressed *TG*, they did not uniformly express genes related to thyroid functionality.  Using pseudotime analysis, doxycycline-responsive cells had two differentiation branches towards non-thyroid lineages and mature thyrocytes.  Dynamics in *TGF-β* pathways and WNT/PCP might play a decisive role in thyrocyte differentiation and maturation  The expression of *TGF-β1*, *TGF-β2* and *TGF-β* *receptor 1* decreased steeply upon maturation of thyrocytes, while *TGF-β* *receptor 2* showed a distinct pattern, which increased during the transition from immature to mature thyrocyte state.  Inhibition of *TGF-β* pathway improved the efficiency of thyroid maturation *in vitro* with increased capacity of iodide uptake and organification. |
| van der Vaart J^65^ | 2021 | Human  Mouse | Thyroid tissue  Thyroid tissue | 3D model  *in vitro* model  Thyroid organoid | Thyroid cells isolation  Thyroid cells culture in BME  Thyroid organoid reconstruction  Viability Assays  Gene expression analysis by qRT-PCR  Bulk mRNAseq data analysis of thyroid organoid  scRNAseq data analysis of thyroid organoid  Immunodetection  Microscopic examination  Thyroid hormone assay | Organoids could be induced in vitro from isolated adult thyroid tissues when plated in proper culture medium with necessary growth factors.  Both murine and human thyroid organoids could be maintained over prolonged culture (>24 passages) without significant change in morphology.  Combinatorial nuclear expression of *NKX2-1* and *PAX8* was confirmed in all cells in the organoids, indicating organoids mainly consisted of expanding populations of thyroid follicular cells.  Both murine and human thyroid organoids showed comparable levels of *TPO*, *PAX8*, and *DUOX2* to primary tissue.  No significant change in expression of stem cell markers was identified in human thyroid organoid with the exception of NGFR, SOX2 and *FOXE1* upregulation.  Nine cell clusters were identified from thyroid-derived organoid through scRNAseq analysis.  Through scRNAseq analysis, thyrocytes from primary thyroid tissue shared high similarity with thyrocytes from cultured organoids.  The lack of a single population and the high comparability between the populations might indicate that thyroid tissue and organoids did not harbor professional stem cells and the growth of organoids might be due to the presence of a transient proliferative cell pool with high resemblance to mature thyroid follicular cells.  Thyroid organoids possessed the thyroid hormone machinery and could secrete thyroid hormones from their basal side.  The cellular structures and features of thyroid organoids were similar to the findings of earlier reports of transmission electron microscopic studies.  Thyroid organoids not only responded to TSH but also responded to *TSHR*ABs derived from serum of a Graves’ disease patient. |
| Liang J^66^ | 2022 | Human | Fetal thyroid tissue | 3D model  *in vitro* model  *in vivo* model  Thyroid organoid | Thyroid follicles and thyrocytes isolation  Cell culture in Matrigel with growth factors  Fetal thyroid organoid culture and maturation  Medium supernatant T4 assay  Gene expression analysis by qRT-PCR  scATACseq data analysis of thyroid organoid  scRNAseq data analysis of thyroid organoid  Colony formation assay and karyotyping  Organoid transplantation into mice  Immunodetection  Microscopic examination | High-resolution atlas of human fetal thyroid glands at 12 and 16 GWs was provided using single cell transcriptome survey.  Thyroid follicular cells began to proliferate rapidly around 12 GWs, and markedly increased in 16 GWs during thyroid development.  Functional analysis revealed that upregulated genes in thyroid follicular cells at 16 GWs were enriched in the thyroid hormone synthesis pathway.  Many signaling pathways were significantly associated with 16GWs upregulated genes, suggesting their roles in thyroid development between 12 to 16 GWs.  A subgroup of more mature follicular cells initiated hormone synthesis from 12 to 16 GWs, indicating thyrocytes cellular heterogeneity existed during development.  Certain groups of potential transcription factors were found to contribute to the differences in the expression of thyroid developments.  Fetal thyroid organoids could be induced in vitro from isolated fetal thyroid tissues when plated in proper culture medium with necessary growth factors.  The fetal thyroid organoid recapitulated canonical follicular morphology and retained the functional ability to produce T4 both in vitro and in NSG mice.  Thyroid organoid formed lager follicular structures in primary culture after 4 days of additional forskolin treatment.  *SLC5A5*, *TSHR*, *TG* and *TPO* expressions were  significantly upregulated after forskolin  treatment with the exception of *NKX2-1* and  *PAX8*.  Transition from fetal thyroid organoid to mature organoid by cAMP induction resembled thyroid development and maturation.  Increasing chromatin accessibility was important to permit transcriptome for fetal thyroid fate determination. |

3D: Three-dimensional

I^131^: Iodide 131

NaI^131^: Radioactive sodium iodide

MIT: Monoiodotyrosine

TSH: Thyrotrophin, Thyroid Stimulating Hormone

DIT: Diiodotyrosine

T4: Thyroxine

T3: Triiodothyronine

FT4: Free thyroxine

FT3: Free triiodothyronine

CAM: Chorioallantoic membrane

AEC: Anterior eye chamber

PGE: Prostaglandins E

PGE1: Prostaglandins E 1

PGE2: Prostaglandins E 2

PY1: 7-oxa-13-prostynoic acid

DBcAMP: Dibutyryl cyclic AMP

APN: Aminopeptidase N

TGE: Thyroid gland equivalent

T78: A cultivated cell strain of rat thyroid cells

FRTL: A cultivated cell strain of rat thyroid cells

FRTL-5: A cultivated cell strain of rat thyroid cells

TG: Thyroglobulin

PTU: Pylthiouracil

EGF: Epidermal growth factor

SCID: Severe combined immunodeficiency

IGF-I: Insulin-like growth factor-I

RCCS: Rotary cell culture system

ECM: Extracellular matrix

BME: Basement membrane extract

KGF: Keratinocyte growth factor

Cx23: Connexin 23

Cx43: Connexin 43

TGF-β: Transforming growth factor-beta

AE: Air exposure

ATP: Adenosine triphosphate

mATP synthase-α: Mitochondrial ATP synthase-α subunit

BrdU: 5-Bromo-2’-deoxyuridine

ESCs: Embryonic stem cells

EBs: Embryonic bodies

SLC5A5: TPO: Na/I symporter

PAX8: Paired box gene 8

TPO: Thyroid peroxidase

TSHR: Thyroid stimulating hormone receptor

TTF2: FOXE1: Forkhead box protein

GFP: Green fluorescent protein

EGFP: Enhanced green fluorescent protein

GFP-Neo^r^: Green fluorescent protein-neomycin-resistant

qRT-PCR: Quantitative reverse transcription-polymerase chain reaction

FACS: Fluoresence-activated cell sorting

SP cells: Side population cells

MP cells:Main population cells

ABCG2: ATP-binding cassette superfamily G member 2

FOX: Forkhead box

sqRT-PCR: Semi-quantitative reverse transcription-polymerase chain reaction

OCT4: Octamer transcription factor 4

TTF1: NKX2-1: NKX2 homeobox 1

RT-PCR: Reverse transcription-polymerase chain reaction

SCs: Stem cells

PSCs: Pluripotent stem cells

iPSCs: Induced pluripotent stem cells

β-Gal: β-galactosidase

SCA1: Stem cell antigen 1

ROSA: Reverse orientation splice acceptor

SOX: Sex-determining region Y-box

BMP: Bone morphogenetic protein

FGF: Fibroblast growth factor

BFGF: Basic fibroblast growth factor

PIPAAm: poly (N-isopropylacrylamide)

LIF: Leukaemia inhibitory factor

MEFs: Mouse embryonic fibroblasts

MB: Mouse embryos

TS: Thyroid spheroids

AS: Allantoic spheroids

ECs: Endothelial cells

VEGFA: Vascular endothelial growth factor A

VEGFR2: Vascular endothelial growth factor receptor 2

LGR4: GPR48: Leucine-rich repeat-containing G-protein-coupled receptor 4

PLO: Poly-L-ornithine hydrochloride

APA: Alginate-PLO-alginate

SDS: Sodium dodecyl sulfate

ADMSCs: Adipose-derived mesenchymal stromal/stem cells

PTH: Parathyroid hormone

Krt7: Keratin 7

Krt14: Keratin 14

TTF: Thyroid transcription factors

siRNA: Small interfering RNA

HHEX: Hematopoietically expressed homeobox protein

DIO2: Deiodinase type 2

TPOCreER2: Tamoxifen induced *TPO*-Cre mice

ROSA26iDTR: Inducible diphtheria toxin receptor homozygous mice

TM: Tamoxifen

DT: Diphtheria toxin

FGF10: Fibroblast growth factor 10

FGFR2b: Fibroblast growth factor receptor 2b

EX1, EX3: Targeted sequence

TGM: Thyroid gland medium

WR: Wnt and R-spondin1

EDU: 5-Ethynyl-2'-deoxyuridine

RNAseq: RNA sequencing

scRNAseq: Single-cell RNA sequencing

DUOX2: Dual Oxidase 2

TSHRABs: TSHR antibodies

ATACseq: Assays for transposase-accessible chromatin using sequencing

GWs: Gestational weeks

NOD: Non obese diabetes

NSG: NOD-SCID IL-2 receptor gamma null

1. Pulvertaft, R.J., Davies, J.R., Weiss, L., and Wilkinson, J.H. (1959). Studies on tissue cultures of human pathological thyroids. The Journal of pathology and bacteriology 77, 19-32. 10.1002/path.1700770103.

2. Pastan, I. (1961). Certain functions of isolated thyroid cells. Endocrinology 68, 924-931. 10.1210/endo-68-6-924.

3. Tong, W., Kerkof, P., and Chaikoff, I.L. (1962). Iodine metabolism of dispersed thyroid cells obtained by trypsinization of sheep thyroid glands. Biochimica et biophysica acta 60, 1-19. 10.1016/0006-3002(62)90365-7.

4. Hung, W., and Winship, T. (1964). IODIDE CONCENTRATING ABILITY OF NORMAL HUMAN THYROID CELLS IN TISSUE CULTURE. Proceedings of the Society for Experimental Biology and Medicine. Society for Experimental Biology and Medicine (New York, N.Y.) 115, 379-381. 10.3181/00379727-115-28918.

5. Kerkof, P.R., Raghupathy, E., and Chaikoff, I.L. (1964). IN VITRO EFFECTS OF THYROTROPIC HORMONE. II. ON UPTAKE AND INCORPORATION OF 131-I INTO IODOAMINO ACIDS BY ISOLATED THYROID CELLS IN MONOLAYER CULTURES. Endocrinology 75, 537-546. 10.1210/endo-75-4-537.

6. Tong, W. (1964). STIMULATORY EFFECT OF THYROTROPIN ON SYNTHESIS OF THYROXINE BY ISOLATED THYROID CELLS. Endocrinology *74*, 304-306. 10.1210/endo-74-2-304.

7. Mallette, J.M., and Anthony, A. (1966). Growth in culture of trypsin dissociated thyroid cells from adult rats. Exp Cell Res *41*, 642-651. 10.1016/s0014-4827(66)80115-5.

8. Shimoda, S.I., Inoue, K., and Greer, M.A. (1966). Inhibition of iodoamino acid synthesis in isolated thyroid epithelial cells by high concentrations of inorganic iodide. Endocrinology *78*, 1171-1176. 10.1210/endo-78-6-1171.

9. Kalderon, A.E., and Wittner, M. (1967). Histochemical studies of thyroid cells in long-term tissue culture. Endocrinology *80*, 797-807. 10.1210/endo-80-5-797.

10. Rodesch, F., and Dumont, J.E. (1967). Metabolic properties of isolated sheep thyroid cells. Exp Cell Res *47*, 386-396. 10.1016/0014-4827(67)90241-8.

11. Hilfer, S.R., Iszard, L.B., and Hilfer, E.K. (1968). Follicle formation in the embryonic chick thyroid. II. Reorganization after dissociation. Zeitschrift fur Zellforschung und mikroskopische Anatomie (Vienna, Austria : 1948) *92*, 256-269. 10.1007/bf00335651.

12. Burke, G., Kowalski, K., and Babiarz, D. (1971). Effects of thyrotropin, prostaglandin E1 and a prostaglandin antago*TPO*t on iodide trapping in isolated thyroid cells. Life sciences. Pt. 2: Biochemistry, general and molecular biology *10*, 513-521. 10.1016/0024-3205(71)90125-1.

13. Fayet, G., Michel-Béchet, M., and Lissitzky, S. (1971). Thyrotrophin-induced aggregation and reorganization into follicles of isolated porcine-thyroid cells in culture. 2. Ultrastructural studies. European journal of biochemistry *24*, 100-111. 10.1111/j.1432-1033.1971.tb19659.x.

14. Rousset, B., Poncet, C., and Mornex, R. (1976). Evidence for a secretion of thyroxine by isolated hog thyroid cells. Biochimica et biophysica acta *437*, 543-561. 10.1016/0304-4165(76)90022-2.

15. Mauchamp, J., Margotat, A., Chambard, M., Charrier, B., Remy, L., and Michel-Bechet, M. (1979). Polarity of three-dimensional structures derived from isolated hog thyroid cells in primary culture. Cell and tissue research *204*, 417-430. 10.1007/bf00233653.

16. Denef, J.F., Björkman, U., and Ekholm, R. (1980). Structural and functional characteristics of isolated thyroid follicles. Journal of ultrastructure research *71*, 185-202. 10.1016/s0022-5320(80)90106-9.

17. Fayet, G., and Hovsépian, S. (1980). In vitro conversion of porcine thyroid cells growing in monolayer into functional follicular cells. Biochimie *62*, 27-32. 10.1016/s0300-9084(80)80367-1.

18. Chambard, M., Gabrion, J., and Mauchamp, J. (1981). Influence of collagen gel on the orientation of epithelial cell polarity: follicle formation from isolated thyroid cells and from preformed monolayers. The Journal of cell biology *91*, 157-166. 10.1083/jcb.91.1.157.

19. Hovsépian, S., Feracci, H., Maroux, S., and Fayet, G. (1982). Kinetic studies of the localization of aminopeptidase N in monolayer and in follicle-associated cultures of porcine thyroid cells. Cell and tissue research *224*, 601-611. 10.1007/bf00213755.

20. Bell, E., Moore, H., Mitchie, C., Sher, S., and Coon, H. (1984). Reconstruction of a thyroid gland equivalent from cells and matrix materials. The Journal of experimental zoology *232*, 277-285. 10.1002/jez.1402320215.

21. Nitsch, L., Tacchetti, C., Tramontano, D., and Ambesi-Impiombato, F.S. (1984). Suspension culture reveals a morphogenetic property of a thyroid epithelial cell line. Experimental cell research *152*, 22-30. 10.1016/0014-4827(84)90226-x.

22. Sho, K., and Kondo, Y. (1984). Insulin modulates thyrotropin-induced follicle reconstruction and iodine metabolism in hog thyroid cells cultured in a chemically defined medium. Biochemical and biophysical research communications *118*, 385-391. 10.1016/0006-291x(84)91314-7.

23. Kitajima, K., Yamashita, K., and Fujita, H. (1985). Fine structural aspects of the shift of zonula occludens and cytoorganelles during the inversion of cell polarity in cultured porcine thyroid follicles. Cell and tissue research *242*, 221-224. 10.1007/bf00225580.

24. Mulcahy, R.T., Rosenkrans, W.A., Jr., Penney, D.P., and Cooper, R.A. (1985). The growth and morphology of FRTL-5 thyroid epithelial cells grown as multicellular spheroids in vitro. In vitro cellular & developmental biology : journal of the Tissue Culture Association *21*, 513-520. 10.1007/bf02620844.

25. Massart, C., Hody, B., Condé, D., Leclech, G., Edan, G., and Nicol, M. (1988). Functional properties of human thyroid follicles cultured within collagen gel. Molecular and cellular endocrinology *56*, 227-234. 10.1016/0303-7207(88)90065-2.

26. Derwahl, M., Studer, H., Huber, G., Gerber, H., and Peter, H.J. (1990). Intercellular propagation of individually programmed growth bursts in FRTL-5 cells. Implications for interpreting growth factor actions. Endocrinology *127*, 2104-2110. 10.1210/endo-127-5-2104.

27. Toda, S., and Sugihara, H. (1990). Reconstruction of thyroid follicles from isolated porcine follicle cells in three-dimensional collagen gel culture. Endocrinology *126*, 2027-2034. 10.1210/endo-126-4-2027.

28. Sasaki, M., Sawada, N., Minase, T., Satoh, M., and Mori, M. (1991). Ultrastructural study of human thyroid cells cultured in collagen cells: Comparison between floating sandwich method and dispersed embedding method. Journal of Clinical Electron Microscopy *22*, 932-933.

29. Westermark, K., Nilsson, M., Ebendal, T., and Westermark, B. (1991). Thyrocyte migration and histiotypic follicle regeneration are promoted by epidermal growth factor in primary culture of thyroid follicles in collagen gel. Endocrinology *129*, 2180-2186. 10.1210/endo-129-4-2180.

30. Martin, A., Valentine, M., Unger, P., Lichtenstein, C., Schwartz, A.E., Friedman, E.W., Shultz, L.D., and Davies, T.F. (1993). Preservation of functioning human thyroid organoids in the scid mouse: 1. System characterization. The Journal of clinical endocrinology and metabolism 77, 305-310. 10.1210/jcem.77.2.8345031.

31. Valentine, M., Martin, A., Unger, P., Katz, N., Shultz, L.D., and Davies, T.F. (1994). Preservation of functioning human thyroid "organoids" in the severe combined immunodeficient mouse. III. Thyrotropin independence of thyroid follicle formation. Endocrinology 134, 1225-1230. 10.1210/endo.134.3.8119163.

32. E, B.-S.M., Reut, B., Gerber, H., Peter, H.J., Paulsson, M., Kaempf, J., Simon, F., Marti, U., Gerber, H., and Bürgi, U. (1998). Alginate gel culture allows the retention of extracellular matrix and follicular structure of rat thyroid tissue but does not lead to the formation of follicles by FRTL-5 cells. Thyroid *8*, 1147-1155. 10.1089/thy.1998.8.1147.

33. Glaser, C., Marti, U., Bürgi-Saville, M.E., Ruchti, C., Gebauer, M., Büchler, M.W., Gerber, H., Bürgi, U., and Peter, H.J. (1999). Inhibition of iodine organification and regulation of follicular size in rat thyroid tissue in vitro. Endocrine *11*, 165-170. 10.1385/endo:11:2:165.

34. Martin, A., Zhou, A., Gordon, R.E., Henderson, S.C., Schwartz, A.E., Friedman, E.W., and Davies, T.F. (2000). Thyroid organoid formation in simulated microgravity: Influence of keratinocyte growth factor. Thyroid *10*, 481-487. 10.1089/thy.2000.10.481.

35. Tonoli, H., Flachon, V., Audebet, C., Callé, A., Jarry-Guichard, T., Statuto, M., Rousset, B., and Munari-Silem, Y. (2000). Formation of three-dimensional thyroid follicle-like structures by polarized FRT cells made communication competent by transfection and stable expression of the connexin-32 gene. Endocrinology *141*, 1403-1413. 10.1210/endo.141.4.7400.

36. Green, L.M., Patel, Z., Murray, D.K., Rightnar, S., Burell, C.G., Gridley, D.S., and Nelson, G.A. (2002). Cytoskeletal and functional changes in bioreactor assembled thyroid tissue organoids exposed to gamma radiation. Journal of radiation research *43 Suppl*, S213-218. 10.1269/jrr.43.s213.

37. Toda, S., Watanabe, K., Yokoi, F., Matsumura, S., Suzuki, K., Ootani, A., Aoki, S., Koike, N., and Sugihara, H. (2002). A new organotypic culture of thyroid tissue maintains three-dimensional follicles with C cells for a long term. Biochem Biophys Res Commun *294*, 906-911. 10.1016/s0006-291x(02)00561-2.

38. Toda, S., Aoki, S., Suzuki, K., Koike, E., Ootani, A., Watanabe, K., Koike, N., and Sugihara, H. (2003). Thyrocytes, but not C cells, actively undergo growth and folliculogenesis at the periphery of thyroid tissue fragments in three-dimensional collagen gel culture. Cell and tissue research *312*, 281-289. 10.1007/s00441-003-0718-0.

39. Lin, R.Y., Kubo, A., Keller, G.M., and Davies, T.F. (2003). Committing embryonic stem cells to differentiate into thyrocyte-like cells in vitro. Endocrinology *144*, 2644-2649. 10.1210/en.2002-0122.

40. Arufe, M.C., Lu, M., Kubo, A., Keller, G., Davies, T.F., and Lin, R.Y. (2006). Directed differentiation of mouse embryonic stem cells into thyroid follicular cells. Endocrinology *147*, 3007-3015. 10.1210/en.2005-1239.

41. Hoshi, N., Kusakabe, T., Taylor, B.J., and Kimura, S. (2007). Side population cells in the mouse thyroid exhibit stem/progenitor cell-like characteristics. Endocrinology *148*, 4251-4258. 10.1210/en.2006-0490.

42. Lan, L., Cui, D., Nowka, K., and Derwahl, M. (2007). Stem cells derived from goiters in adults form spheres in response to intense growth stimulation and require thyrotropin for differentiation into thyrocytes. J Clin Endocrinol Metab *92*, 3681-3688. 10.1210/jc.2007-0281.

43. Fierabracci, A., Puglisi, M.A., Giuliani, L., Mattarocci, S., and Gallinella-Muzi, M. (2008). Identification of an adult stem/progenitor cell-like population in the human thyroid. The Journal of endocrinology *198*, 471-487. 10.1677/joe-07-0552.

44. Arauchi, A., Shimizu, T., Yamato, M., Obara, T., and Okano, T. (2009). Tissue-engineered thyroid cell sheet rescued hypothyroidism in rat models after receiving total thyroidectomy comparing with nontransplantation models. Tissue Eng Part A *15*, 3943-3949. 10.1089/ten.TEA.2009.0119.

45. Antonica, F., Kasprzyk, D.F., Opitz, R., Iacovino, M., Liao, X.H., Dumitrescu, A.M., Refetoff, S., Peremans, K., Manto, M., Kyba, M., and Costagliola, S. (2012). Generation of functional thyroid from embryonic stem cells. Nature *491*, 66-71. 10.1038/nature11525.

46. Ozaki, T., Matsubara, T., Seo, D., Okamoto, M., Nagashima, K., Sasaki, Y., Hayase, S., Murata, T., Liao, X.H., Hanson, J., et al. (2012). Thyroid regeneration: characterization of clear cells after partial thyroidectomy. Endocrinology *153*, 2514-2525. 10.1210/en.2011-1365.

47. Ma, R., Latif, R., and Davies, T.F. (2013a). Thyroid follicle formation and thyroglobulin expression in multipotent endodermal stem cells. Thyroid *23*, 385-391. 10.1089/thy.2012.0644.

48. Okamoto, M., Hayase, S., Miyakoshi, M., Murata, T., and Kimura, S. (2013). Stem cell antigen 1-positive mesenchymal cells are the origin of follicular cells during thyroid regeneration. PloS one *8*, e80801. 10.1371/journal.pone.0080801.

49. Kurmann, A.A., Serra, M., Hawkins, F., Rankin, S.A., Mori, M., Astapova, I., Ullas, S., Lin, S., Bilodeau, M., Rossant, J., et al. (2015). Regeneration of Thyroid Function by Transplantation of Differentiated Pluripotent Stem Cells. Cell stem cell *17*, 527-542. 10.1016/j.stem.2015.09.004.

50. Ma, R., Latif, R., and Davies, T.F. (2015a). Human embryonic stem cells form functional thyroid follicles. Thyroid *25*, 455-461. 10.1089/thy.2014.0537.

51. Ma, R., Morshed, S. A., Latif, R., Davies, T. F.. Thyroid cell differentiation from murine induced pluripotent stem cells. *Front Endocrinol (Lausanne)* **2015,** *6*, 56.

52. Antonica, F., Kasprzyk, D.F., Schiavo, A.A., Romitti, M., and Costagliola, S. (2017). Generation of Functional Thyroid Tissue Using 3D-Based Culture of Embryonic Stem Cells. Methods Mol Biol *1597*, 85-95. 10.1007/978-1-4939-6949-4_7.

53. Bulanova, E.A., Koudan, E.V., Degosserie, J., Heymans, C., Pereira, F.D., Parfenov, V.A., Sun, Y., Wang, Q., Akhmedova, S.A., Sviridova, I.K., et al. (2017). Bioprinting of a functional vascularized mouse thyroid gland construct. Biofabrication *9*, 034105. 10.1088/1758-5090/aa7fdd.

54. Lee, J., Yi, S., Chang, J.Y., Kang, Y.E., Kim, H.J., Park, K.C., Yang, K.J., Sul, H.J., Kim, J.O., Yi, H.S., et al. (2017). Regeneration of thyroid follicles from primordial cells in a murine thyroidectomized model. Laboratory investigation; a journal of technical methods and pathology *97*, 478-489. 10.1038/labinvest.2016.158.

55. Yang, Y., Opara, E.C., Liu, Y., Atala, A., and Zhao, W. (2017). Microencapsulation of porcine thyroid cell organoids within a polymer microcapsule construct. Experimental biology and medicine (Maywood, N.J.) *242*, 286-296. 10.1177/1535370216673746.

56. Saito, Y., O*TPO*hi, N., Takami, H., Seishima, R., Inoue, H., Hirata, Y., Kameyama, K., Tsuchihashi, K., Sugihara, E., Uchino, S., et al. (2018). Development of a functional thyroid model based on an organoid culture system. Biochem Biophys Res Commun *497*, 783-789. 10.1016/j.bbrc.2018.02.154.

57. Alfieri, M., Barbaro, F., Consolini, E., Bassi, E., Dallatana, D., Bergonzi, C., Bianchera, A., Bettini, R., Toni, R., and Elviri, L. (2019). A targeted mass spectrometry method to screen collagen types I-V in the decellularized 3D extracellular matrix of the adult male rat thyroid. Talanta *193*, 1-8. 10.1016/j.talanta.2018.09.087.

58. Pan, J., Li, H., Fang, Y., Shen, Y.B., Zhou, X.Y., Zhu, F., Zhu, L.X., Du, Y.H., Yu, X.F., Wang, Y., et al. (2019). Regeneration of a Bioengineered Thyroid Using Decellularized Thyroid Matrix. Thyroid *29*, 142-152. 10.1089/thy.2018.0068.

59. Deisenroth, C., Soldatow, V.Y., Ford, J., Stewart, W., Brinkman, C., LeCluyse, E.L., MacMillan, D.K., and Thomas, R.S. (2020). Development of an In Vitro Human Thyroid Microtissue Model for Chemical Screening. Toxicol Sci *174*, 63-78. 10.1093/toxsci/kfz238.

60. Jang, D., Marcus-Samuels, B., Morgan, S.J., Klubo-Gwiezdzinska, J., Neumann, S., and Gershengorn, M.C. (2020). Thyrotropin regulation of differentiated gene transcription in adult human thyrocytes in primary culture. Mol Cell Endocrinol *518*, 111032. 10.1016/j.mce.2020.111032.

61. Ran, Q., Zhou, Q., Oda, K., Yasue, A., Abe, M., Ye, X., Li, Y., Sasaoka, T., Sakimura, K., Ajioka, Y., and Saijo, Y. (2020). Generation of Thyroid Tissues From Embryonic Stem Cells via Blastocyst Complementation In Vivo. Front Endocrinol (Lausanne) *11*, 609697. 10.3389/fendo.2020.609697.

62. Ma, R., Morshed, S.A., Latif, R., and Davies, T.F. (2021). A Stem Cell Surge During Thyroid Regeneration. Front Endocrinol (Lausanne) *11*, 606269. 10.3389/fendo.2020.606269.

63. Ogundipe, V.M.L., Groen, A.H., Hosper, N., Nagle, P.W.K., Hess, J., Faber, H., Jellema, A.L., Baanstra, M., Links, T.P., Unger, K., et al. (2021). Generation and Differentiation of Adult Tissue-Derived Human Thyroid Organoids. Stem Cell Reports *16*, 913-925. 10.1016/j.stemcr.2021.02.011.

64. Romitti, M., Eski, S.E., Fonseca, B.F., Gillotay, P., Singh, S.P., and Costagliola, S. (2021b). Single-Cell Trajectory Inference Guided Enhancement of Thyroid Maturation In Vitro Using *TG*F-Beta Inhibition. Front Endocrinol (Lausanne) *12*, 657195. 10.3389/fendo.2021.657195.

65. van der Vaart, J., Bosmans, L., Sijbesma, S.F., Knoops, K., van de Wetering, W.J., Otten, H.G., Begthel, H., Borel Rinkes, I.H.M., Korving, J., Lentjes, E., et al. (2021). Adult mouse and human organoids derived from thyroid follicular cells and modeling of Graves' hyperthyroidism. Proc Natl Acad Sci U S A *118*. 10.1073/pnas.2117017118.

66. Liang, J., Qian, J., Yang, L., Chen, X., Wang, X., Lin, X., Wang, X., and Zhao, B. (2022). Modeling Human Thyroid Development by Fetal Tissue-Derived Organoid Culture. Adv Sci (Weinh) *9*, e2105568. 10.1002/advs.202105568.
